# Correction: Bacterial assemblages on eggs reflect nesting strategies in wetland-associated birds

**DOI:** 10.1371/journal.pone.0341220

**Published:** 2026-01-20

**Authors:** Wouter F. D. van Dongen, Hanja B. Brandl, Alžbeta Darolová, Ján Krištofík, Herbert Hoi

An additional affiliation is missing for the second author. Hanja B. Brandl is also affiliated with Department of Collective Behaviour, Max Planck Institute of Animal Behavior, Konstanz, Germany and Centre for the Advanced Study of Collective Behaviour, University of Konstanz, Konstanz, Germany.
