# Supplementary material for: Bacterial assemblages on eggs reflect nesting strategies in wetland-associated birds
Source: PLoS One. 2025 Sep 17;20(9):e0332380. doi: 10.1371/journal.pone.0332380 (PMC12443268; doi:10.1371/journal.pone.0332380)
Supplement: S1 Fig — Species are a) great-crested grebe (wet-nester), b) common coot (dry-nester) and c) mute swan (dry-nester). (DOCX) [file pone.0332380.s001.docx]

**S1 Fig.** **Wet and dry nests located at the study sites.** Species are a) great-crested grebe (wet-nester), b) common coot (dry-nester) and c) mute swan (dry-nester)
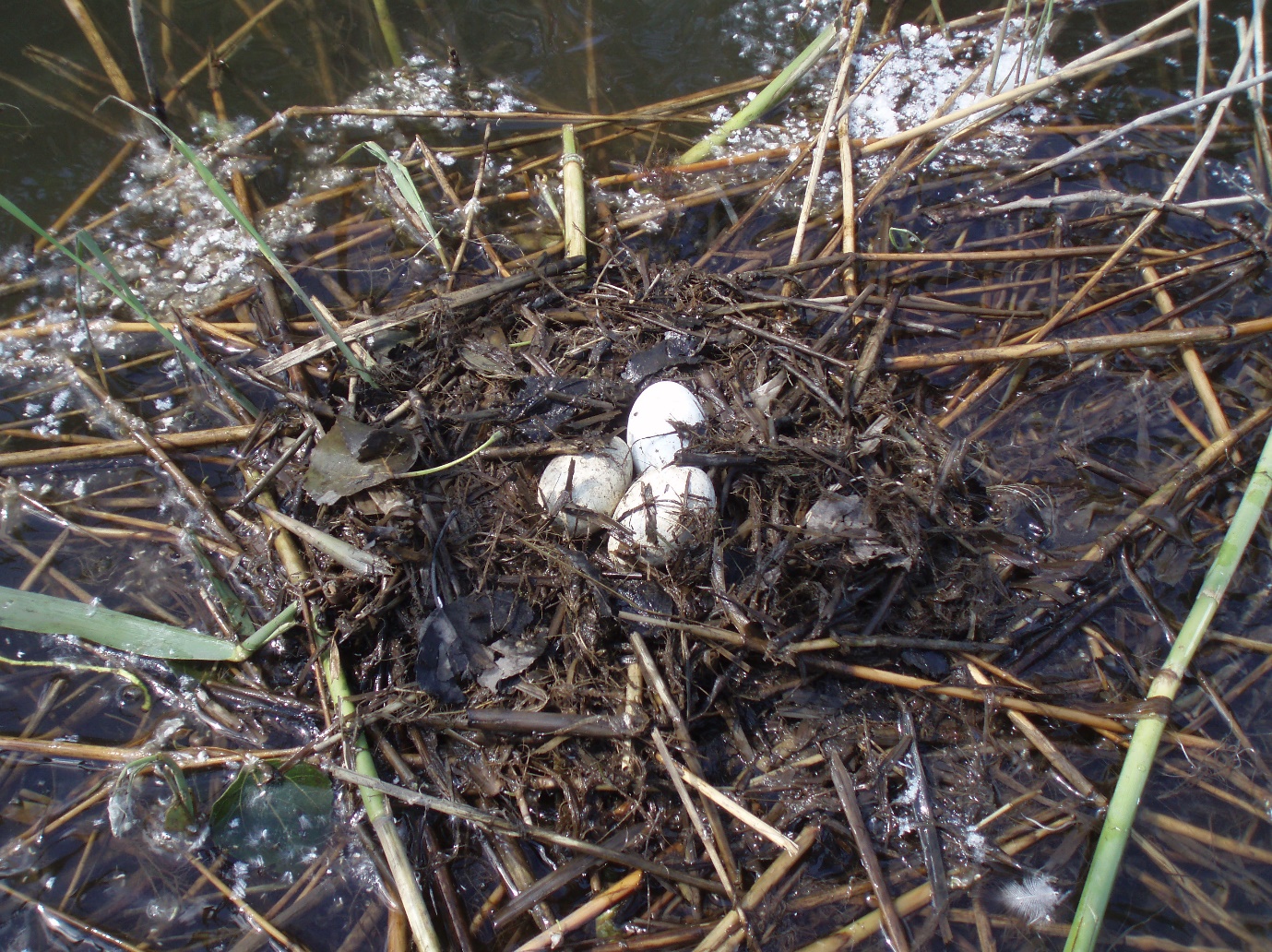


**Fig 1a**


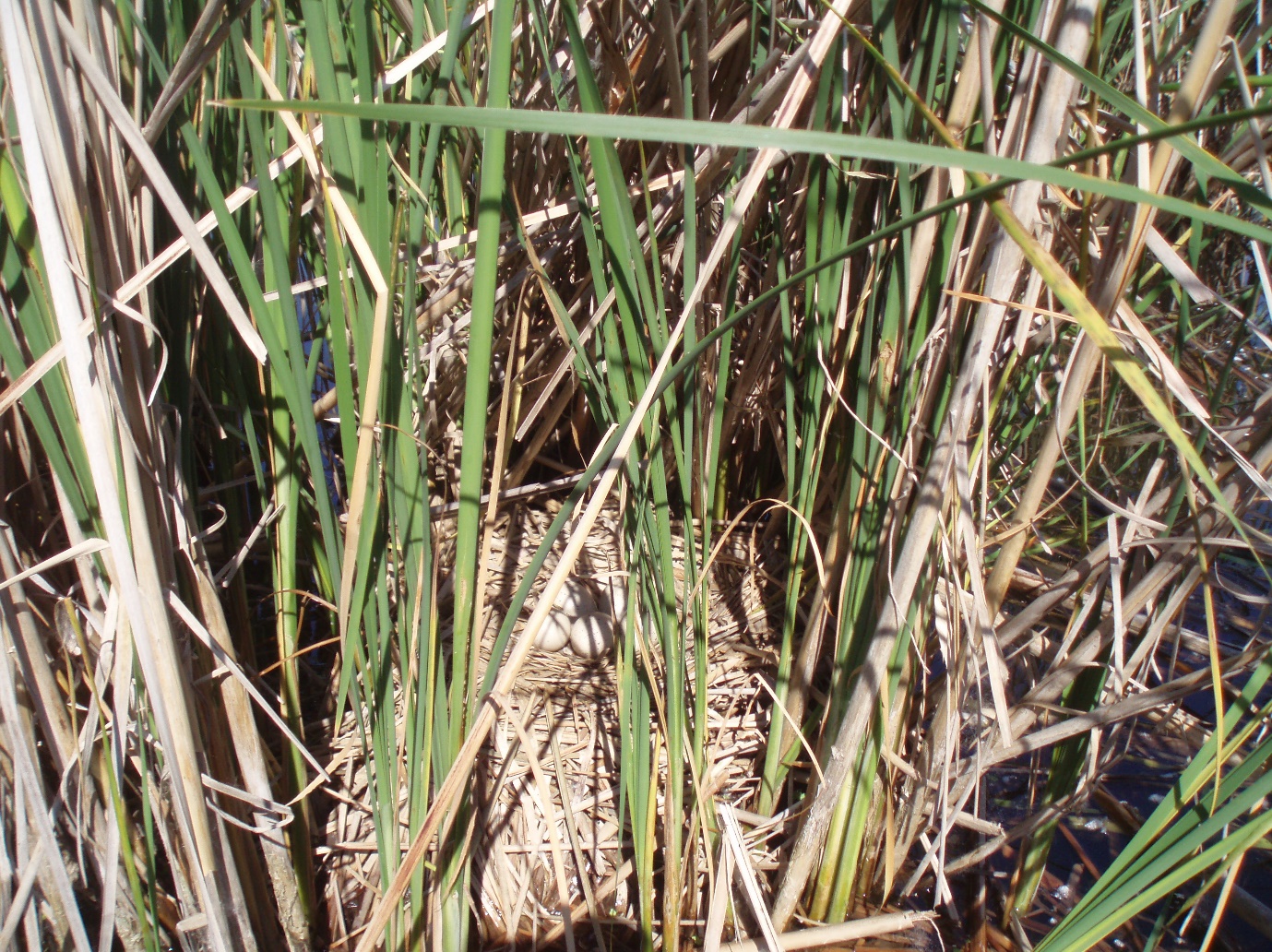


**Fig 1b**

**
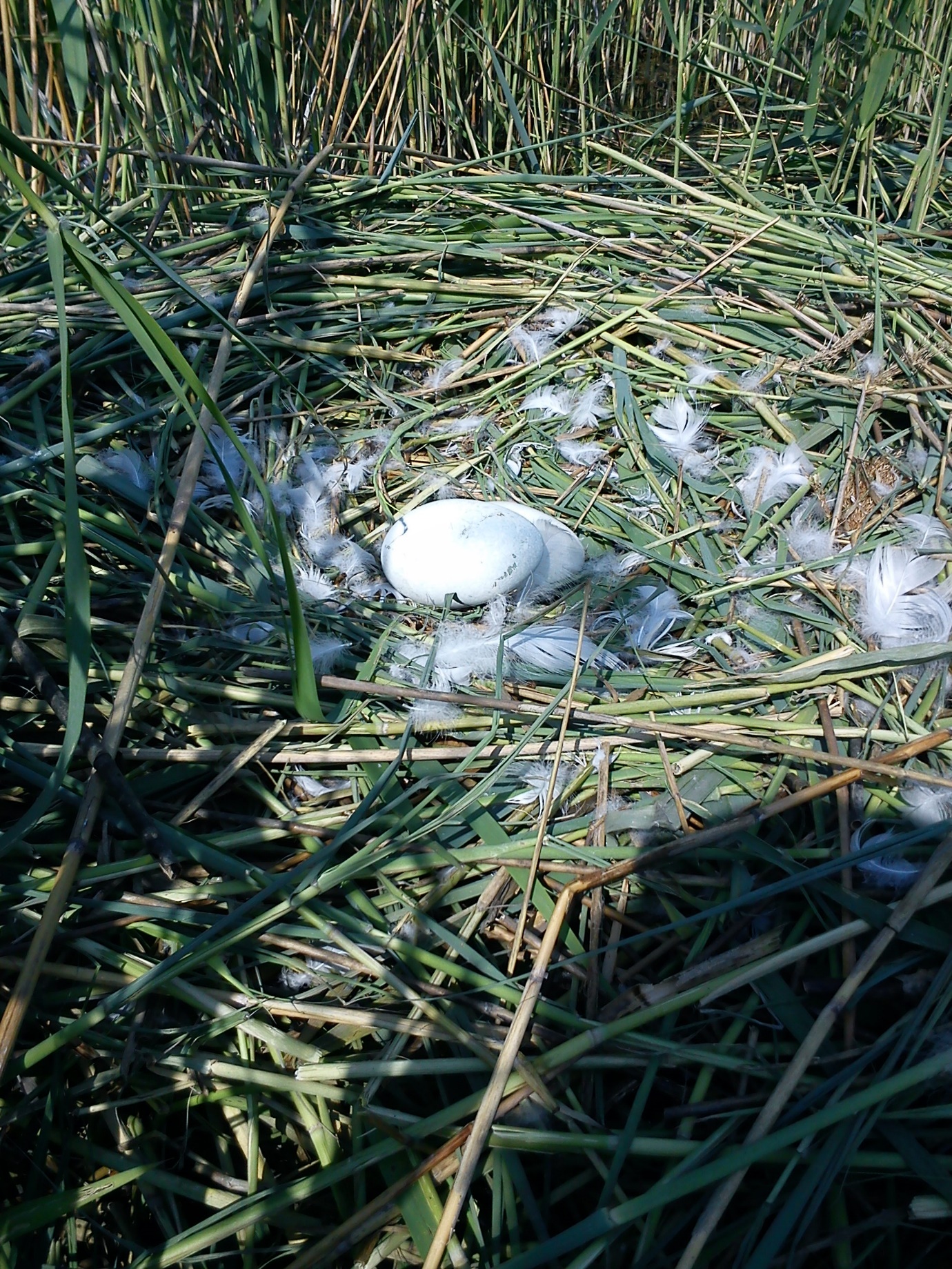
**

**Fig 1c**
