# Supplementary material for: Bacterial assemblages on eggs reflect nesting strategies in wetland-associated birds
Source: PLoS One. 2025 Sep 17;20(9):e0332380. doi: 10.1371/journal.pone.0332380 (PMC12443268; doi:10.1371/journal.pone.0332380)
Supplement: S2 Table — Samples sizes include number of nests sampled, samples used for bacterial culture and samples used for ARISA analysis. (DOCX) [file pone.0332380.s002.docx]

**S2 Table. Sample sizes for each study species and environmental water.** Samples sizes include number of nests sampled, samples used for bacterial culture and samples used for ARISA analysis.

| **Species** | **Scientific name** | **Nest-type** | **Number of nests** | **Culture samples** | **ARISA samples** |
| --- | --- | --- | --- | --- | --- |
| Great-crested grebe | *Podiceps cristatus* | Wet | 43 | 43 | 22 |
| Little grebe | *Tachybaptus ruficollis* | Wet | 8 | 8 | 4 |
| Mallard | *Anas platyrhynchos* | Dry | 2 | 2 | 2 |
| Pochard | *Aythya ferina* | Dry | 5 | 5 | 5 |
| Red-crested pochard | *Netta rufina* | Dry | 1 | 1 | 1 |
| Greylag goose | *Anser anser* | Dry | 3 | 3 | 3 |
| Mute swan | *Cygnus olor* | Dry | 4 | 4 | 4 |
| Common coot | *Fulica atra* | Dry | 28 | 28 | 25 |
| Moorhen | *Gallinula chloropus* | Dry | 1 | 1 | 1 |
| Marsh harrier | *Circus aeruginosus* | Dry | 1 | 1 | 1 |
| Little bittern | *Ixobrychus minutus* | Dry | 1 | 1 | 1 |
| Purple heron | *Ardea purpurea* | Dry | 1 | 1 | 1 |
| Savi´s warbler | *Locustella luscinioides* | Dry | 3 | 3 | 3 |
| Water | N/A | N/A | 24 | 24 | 22 |
|  |  |  |  |  |  |
|  |  | Total | 125 | 125 | 95 |
