# Supplementary material for: Bacterial assemblages on eggs reflect nesting strategies in wetland-associated birds
Source: PLoS One. 2025 Sep 17;20(9):e0332380. doi: 10.1371/journal.pone.0332380 (PMC12443268; doi:10.1371/journal.pone.0332380)
Supplement: S3 Table — Statistics include sample size (N), mean and standard deviation (X̅ ± SD) and Range (minimum – maximum). Sample types are wet-nester (WN), dry-nester (DN) and water sample (WS). (DOCX) [file pone.0332380.s003.docx]

**S3 Table.** **Descriptive statistics of cultured bacteria across thirteen study species and water samples.** Statistics include sample size (N), mean and standard deviation (X̅±SD) and Range (minimum – maximum). Sample types are wet-nester (WN), dry-nester (DN) and water sample (WS).

|  |  |  | Haemolytic bacteria | | Non-haemolytic bacteria | |
| --- | --- | --- | --- | --- | --- | --- |
| Species | Sample type | N | X̅±SD | Range | X̅±SD | Range |
| Great-crested grebe | WN | 43 | 9 762 488 ± 12 788 564 | 0 – 57 500 000 | 42 906 476 ± 78 007 120 | 15 000 – 455 000 000 |
| Little grebe | WN | 8 | 3 879 875 ± 6 869 623 | 7000 – 16 250 000 | 17 486 812 ± 29 662 743 | 4000 – 68 375 000 |
| Common coot | DN | 28 | 4536 ± 12 276 | 0 – 48 000 | 48 800 ± 157 892 | 180 – 818 000 |
| Greylag goose | DN | 3 | 1000 ± 1732 | 0 – 3000 | 1 721 466 ± 2 194 125 | 27 400 – 4 200 000 |
| Little bittern | DN | 1 | 0 | 0 | 2700 | 2700 |
| Mallard | DN | 2 | 5040 ± 7015 | 80 – 10 000 | 666 035 ± 938 988 | 2070 –1 330 000 |
| Marsh harrier | DN | 1 | 0 | 0 | 1 130 000 | 1 130 000 |
| Moorhen | DN | 1 | 0 | 0 | 44 000 | 44 000 |
| Mute swan | DN | 4 | 800 ± 1534 | 0 – 3100 | 2 290 800 ± 4 080 234 | 6300 – 8 400 000 |
| Pochard | DN | 5 | 80 520 ± 178 596 | 0 – 400 000 | 1 471 500 ± 2 934 616 | 1900 – 6 700 000 |
| Purple heron | DN | 1 | 0 | 0 | 40 | 40 |
| Red-crested pochard | DN | 1 | 110 | 110 | 700 | 700 |
| Savi´s warbler | DN | 3 | 17 ± 15 | 0 – 30 | 220 ± 206 | 50 – 450 |
| Water | WS | 24 | 2082 ± 8941 | 0 – 44 000 | 17 542 ± 67 293 | 40 – 329 680 |

|  |  |  | *Enterococcus* sp. | | Coliforms | |
| --- | --- | --- | --- | --- | --- | --- |
| Species | Sample type | N | X̅±SD | Range | X̅±SD | Range |
| Great-crested grebe | WN | 43 | 4 993 866 ± 9 418 144 | 0 – 420 500 000 | 4 638 052 ± 7 450 252 | 0 – 28 000 000 |
| Little grebe | WN | 8 | 2 135 712 ± 3 857 258 | 0 – 8 750 000 | 1 384 312 ± 2 976 668 | 0 – 8 500 000 |
| Common coot | DN | 28 | 21 202 ± 68 639 | 0 – 320 000 | 3417 ± 13 383 | 0 – 70 000 |
| Greylag goose | DN | 3 | 230 566 ± 284 908 | 2700 – 550 000 | 12 333 ± 15 695 | 0 – 30 000 |
| Little bittern | DN | 1 | 0 | 0 | 200 | 200 |
| Mallard | DN | 2 | 460 210 ± 650 241 | 420 – 920 000 | 260 000 ± 367 695 | 0 – 520 000 |
| Marsh harrier | DN | 1 | 830 000 | 830 000 | 0 | 0 |
| Moorhen | DN | 1 | 25 500 | 25 500 | 1800 | 1800 |
| Mute swan | DN | 4 | 122 125 ± 188 673 | 1500 – 400 000 | 1 377 500 ± 2 682 167 | 0 – 5 400 000 |
| Pochard | DN | 5 | 265 520 ± 524 346 | 400 – 1 200 000 | 0 | 0 |
| Purple heron | DN | 1 | 370 | 370 | 0 | 0 |
| Red-crested pochard | DN | 1 | 510 | 510 | 10 | 10 |
| Savi´s warbler | DN | 3 | 7 ± 11 | 0 – 20 | 0 | 0 |
| Water | WS | 24 | 101 ± 149 | 0 – 570 | 58 ± 182 | 0 – 900 |

| Species |  |  | *Staphylococcus/Streptococcus* sp. | |
| --- | --- | --- | --- | --- |
|  | Sample type | N | X̅±SD | Range |
| Great-crested grebe | WN | 43 | 2 237 959 ± 4 016 430 | 0 – 2 125 000 |
| Little grebe | WN | 8 | 2 077 000 ± 3 549 593 | 0 – 8 125 000 |
| Common coot | DN | 28 | 29 702 ± 62 162 | 0 – 230 000 |
| Greylag goose | DN | 3 | 466 600 ± 714 843 | 4800 – 1 290 000 |
| Little bittern | DN | 1 | 6600 | 6600 |
| Mallard | DN | 2 | 277 500 ± 385 373 | 5000 – 555 000 |
| Marsh harrier | DN | 1 | 60 000 | 60 000 |
| Moorhen | DN | 1 | 1200 | 1200 |
| Mute swan | DN | 4 | 1 321 850 ± 2 585 509 | 4400 – 5 200 000 |
| Pochard | DN | 5 | 436 800 ± 626 770 | 0 – 1 500 000 |
| Purple heron | DN | 1 | 10 | 10 |
| Red-crested pochard | DN | 1 | 50 | 50 |
| Savi´s warbler | DN | 3 | 0 | 0 |
| Water | WS | 24 | 2452 ± 9759 | 0 – 48 000 |
