# Supplementary material for: Bacterial assemblages on eggs reflect nesting strategies in wetland-associated birds
Source: PLoS One. 2025 Sep 17;20(9):e0332380. doi: 10.1371/journal.pone.0332380 (PMC12443268; doi:10.1371/journal.pone.0332380)
Supplement: S4 Table — Separate GLMMs were conducted for each bacterial taxon. Coefficients for the great-crested grebe were set to zero. (DOCX) [file pone.0332380.s004.docx]

**S4 Table.** **Generalised linear mixed model output on the effect of species (common coot [dry-nester] and great-crested grebe [wet-nester]), number of eggs in nest and sampling data on the abundance of colony forming units found on eggshells.** Separate GLMMs were conducted for each bacterial taxon. Coefficients for the great-crested grebe were set to zero.

| **Model term** | **Coefficient** | **t** | **P** |
| --- | --- | --- | --- |
| Haemolytic bacteria | | | |
| Intercept | 1.830±0.180 | 10.172 | <0.001 |
| Species | -0.814±0.095/0 | -8.592 | <0.001 |
| Number eggs | -0.022±0.016 | -1.358 | 0.180 |
| Date | 0.001±0.002 | 0.632 | 0.530 |
| Non-haemolytic bacteria | | | |
| Intercept | 1.804±0.119 | 15.118 | <0.001 |
| Species | -0.568±0.063/0 | -9.049 | <0.001 |
| Number eggs | 0.004±0.010 | 0.365 | 0.716 |
| Date | 0.001±0.001 | 1.038 | 0.303 |
| *Enterococcus* sp. | | | |
| Intercept | 1.542±0.226 | 6.838 | <0.001 |
| Species | -0.656±0.118/0 | -5.559 | <0.001 |
| Number eggs | 0.027±0.019 | 1.411 | 0.163 |
| Date | 0.000±0.002 | 0.043 | 0.965 |
| Coliforms | | | |
| Intercept | 9.501±1.665 | 5.707 | <0.001 |
| Species | -3.655±0.743/0 | -4.917 | <0.001 |
| Number eggs | 0.017±0.119 | 0.142 | 0.888 |
| Date | 0.050±0.015 | 3.303 | 0.002 |
| *Staphylococcus/Streptococcus* sp. | | | |
| Intercept | 14.600±1.363 | 10.714 | <0.001 |
| Species | -4.586±0.643/0 | -7.138 | <0.001 |
| Number eggs | -0.031±0.095 | -0.327 | 0.745 |
| Date | 0.000±0.014 | 0.020 | 0.984 |
