# Supplementary material for: Bacterial assemblages on eggs reflect nesting strategies in wetland-associated birds
Source: PLoS One. 2025 Sep 17;20(9):e0332380. doi: 10.1371/journal.pone.0332380 (PMC12443268; doi:10.1371/journal.pone.0332380)
Supplement: S5 Fig — Species are a) common coot (n = 27), b) moorhen (n = 1), c) pochard (n = 5), d) Eurasian marsh harrier (n = 1), e) great-crested grebe (n = 29), f) greylag goose (n = 3), g) little bittern (n = 1), h) little grebe (n = 4), i) mallard (n = 2), j) mute swan (n = 4), k) purple heron (n = 1), l) red-crested pochard (n = 1), m) Savi’s warbler (n = 3), n) water (n = 22). Prevalence was calculated as the percent of samples in which each OTU was detected. Each vertical bar represents an OTU and the position of each OTU is identical within each graph. (DOCX) [file pone.0332380.s005.docx]

**S5 Fig.** **Prevalence of OTUs found on eggs of different bird species and in water samples**. Species are a) common coot (n = 27), b) common moorhen (n = 1), c) common pochard (n = 5), d) Eurasian marsh harrier (n = 1), e) great-crested grebe (n = 29), f) greylag goose (n = 3), g) little bittern (n = 1), h) little grebe (n = 4), i) mallard (n = 2), j) mute swan (n = 4), k) purple heron (n = 1), l) red-crested pochard (n = 1), m) Savi’s warbler (n = 3), n) water (n = 22). Prevalence was calculated as the percent of samples in which each OTU was detected. Each vertical bar represents an OTU and the position of each OTU is identical within each graph.

**
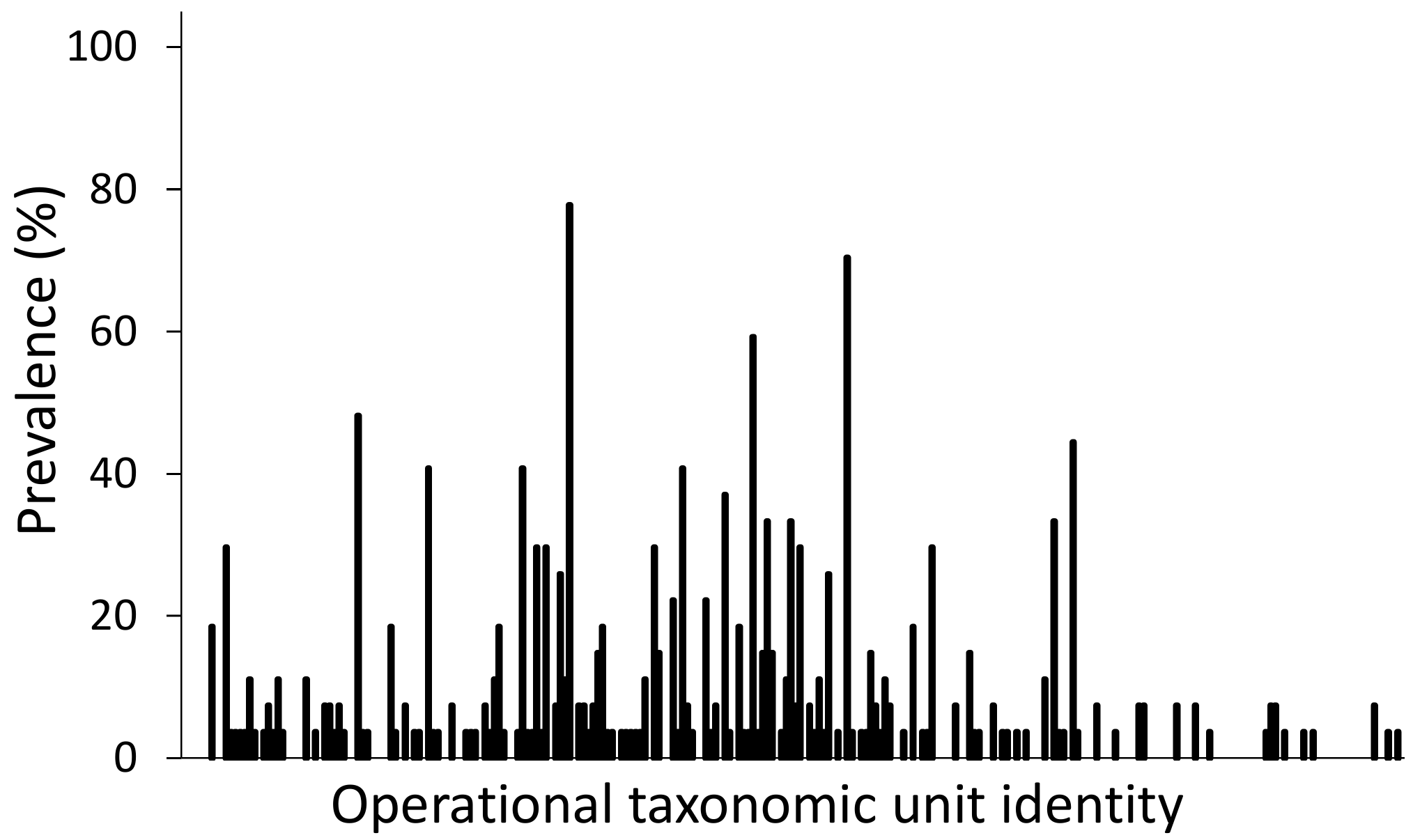
**

**Fig 5a. (common coot)**

**
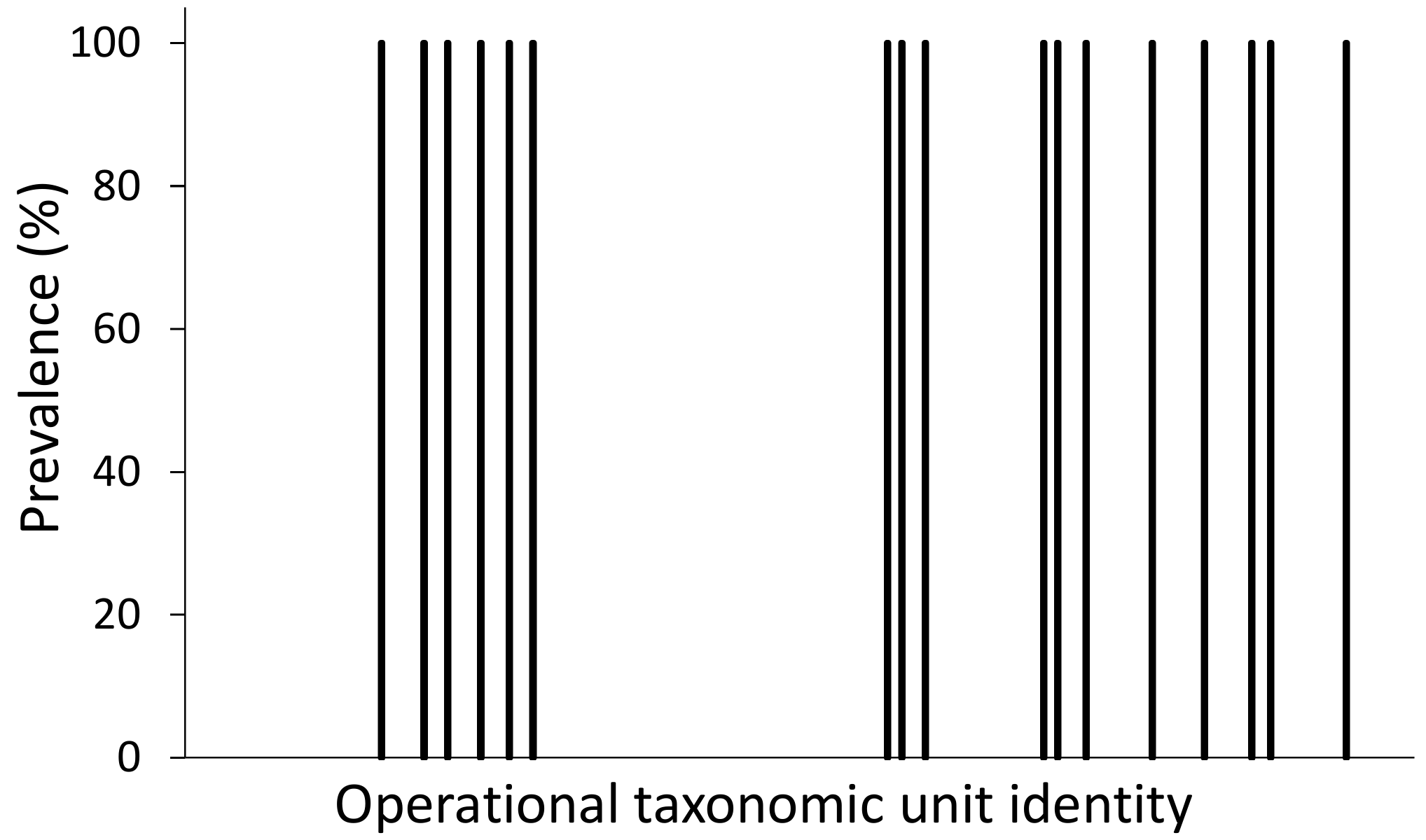
**

**Fig 5b. (common moorhen)**

**
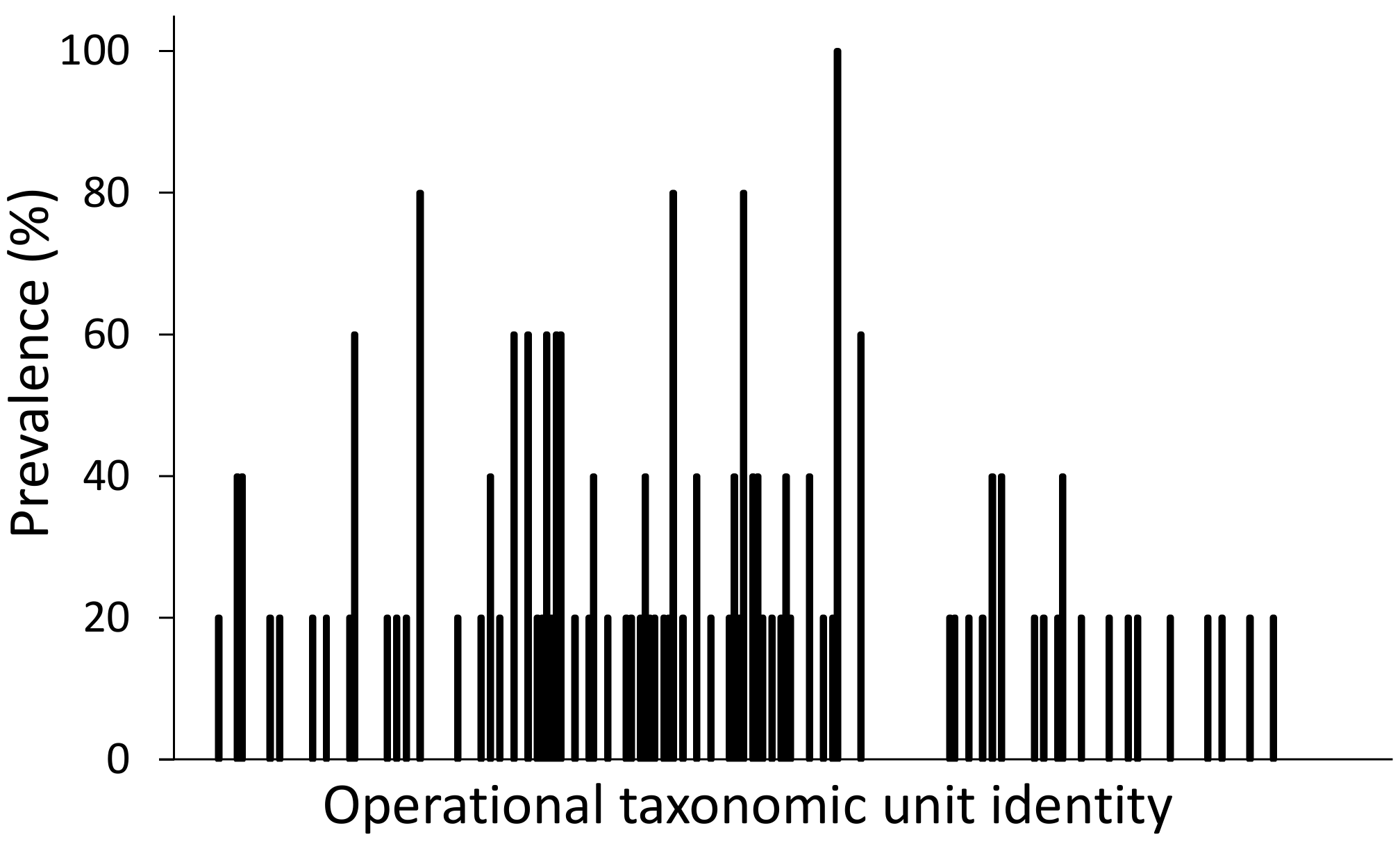
**

**Fig 5c. (common pochard)**

**
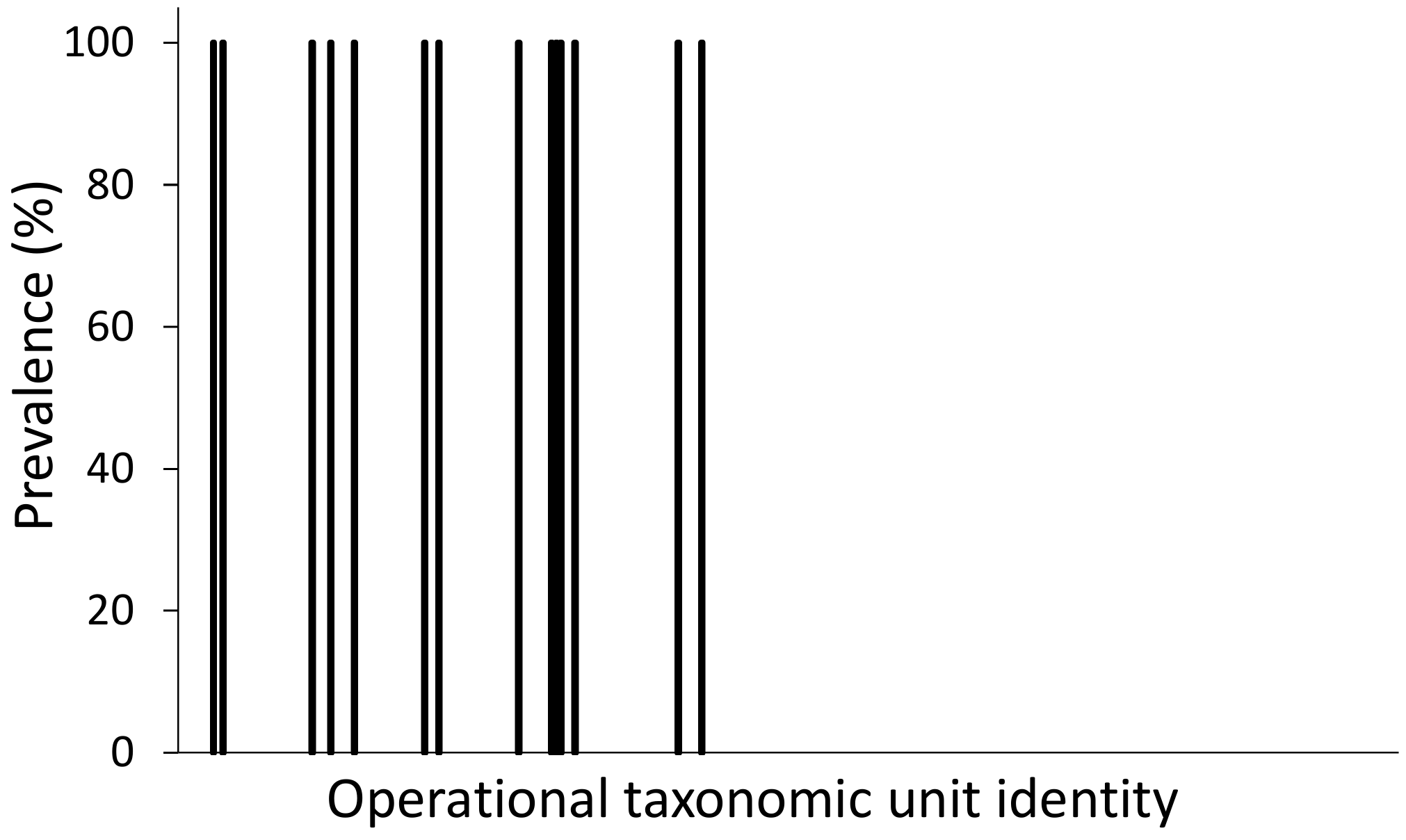
**

**Fig 5d. (Eurasian marsh harrier)**

**
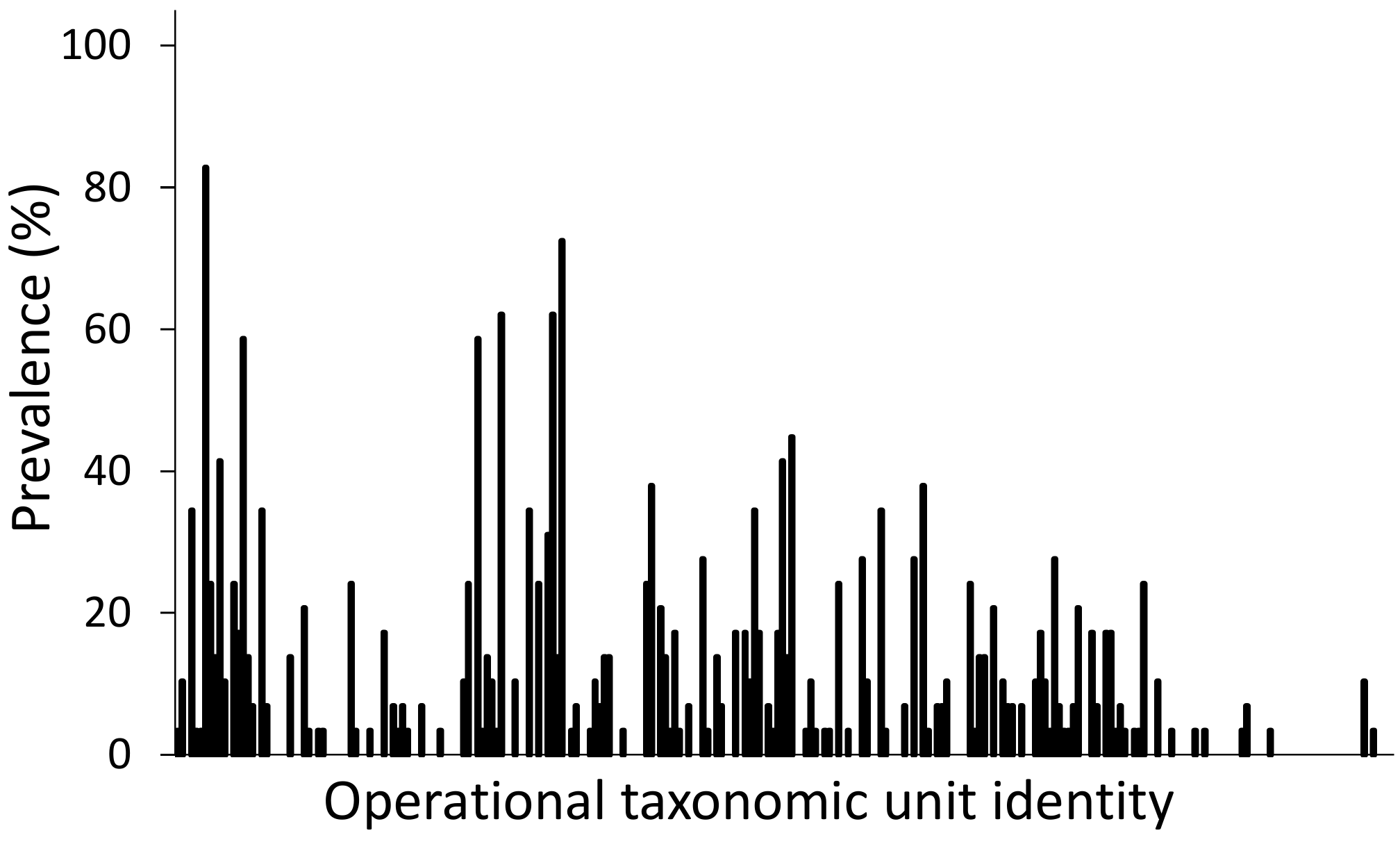
**

**Fig 5e. (great-crested grebe)**

**
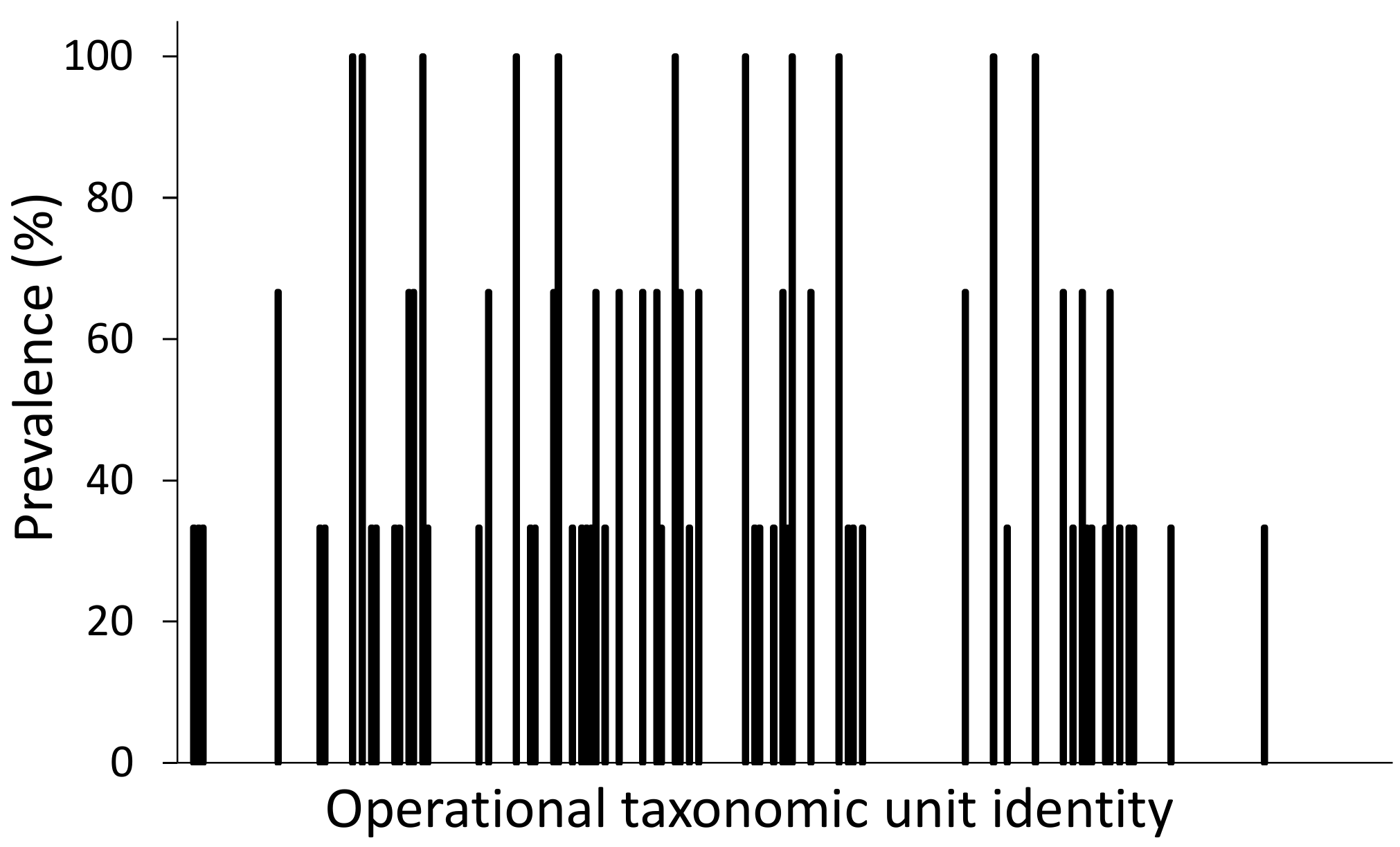
**

**Fig 5f. (greylag goose)**

**
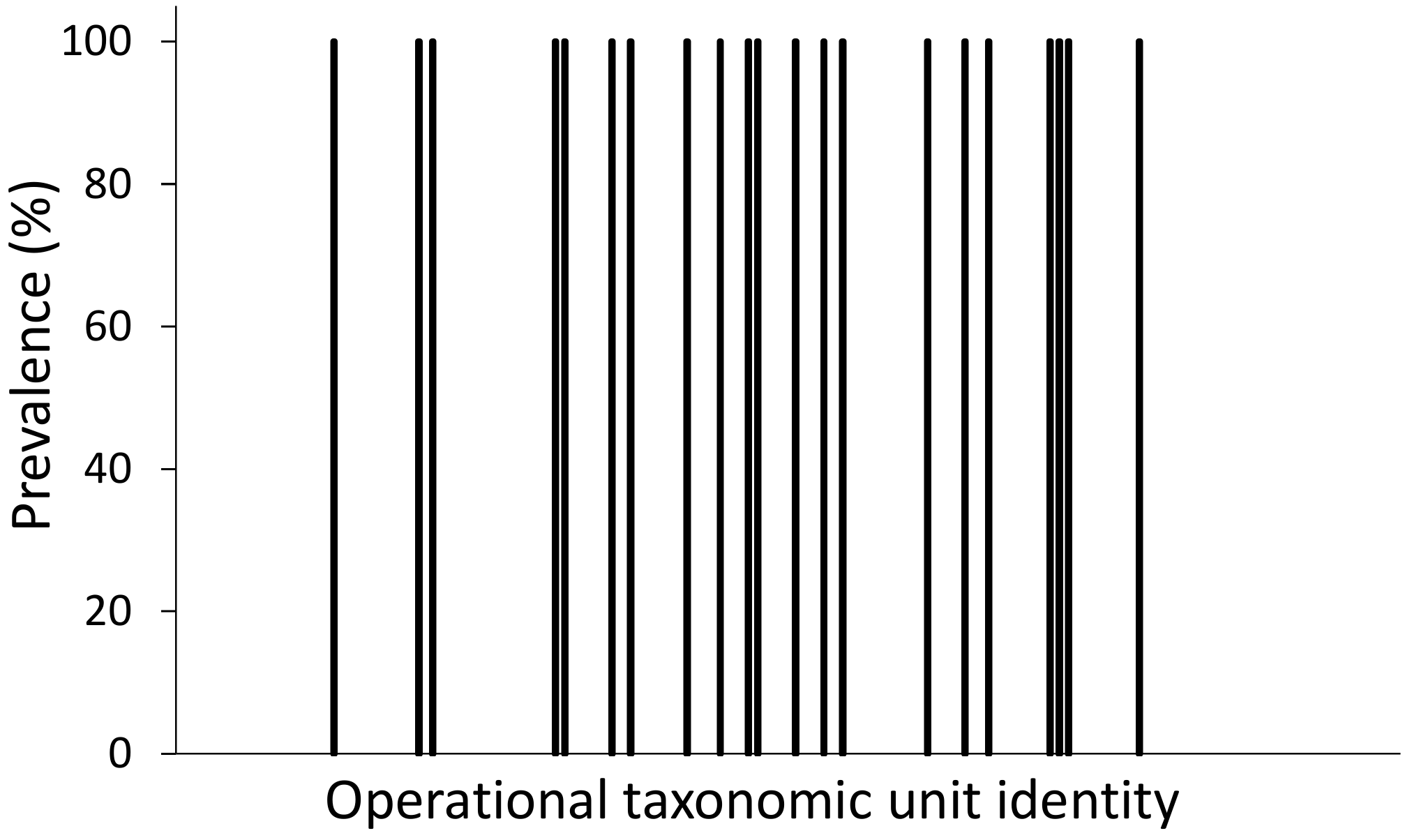
**

**Fig 5g. (little bittern)**

**
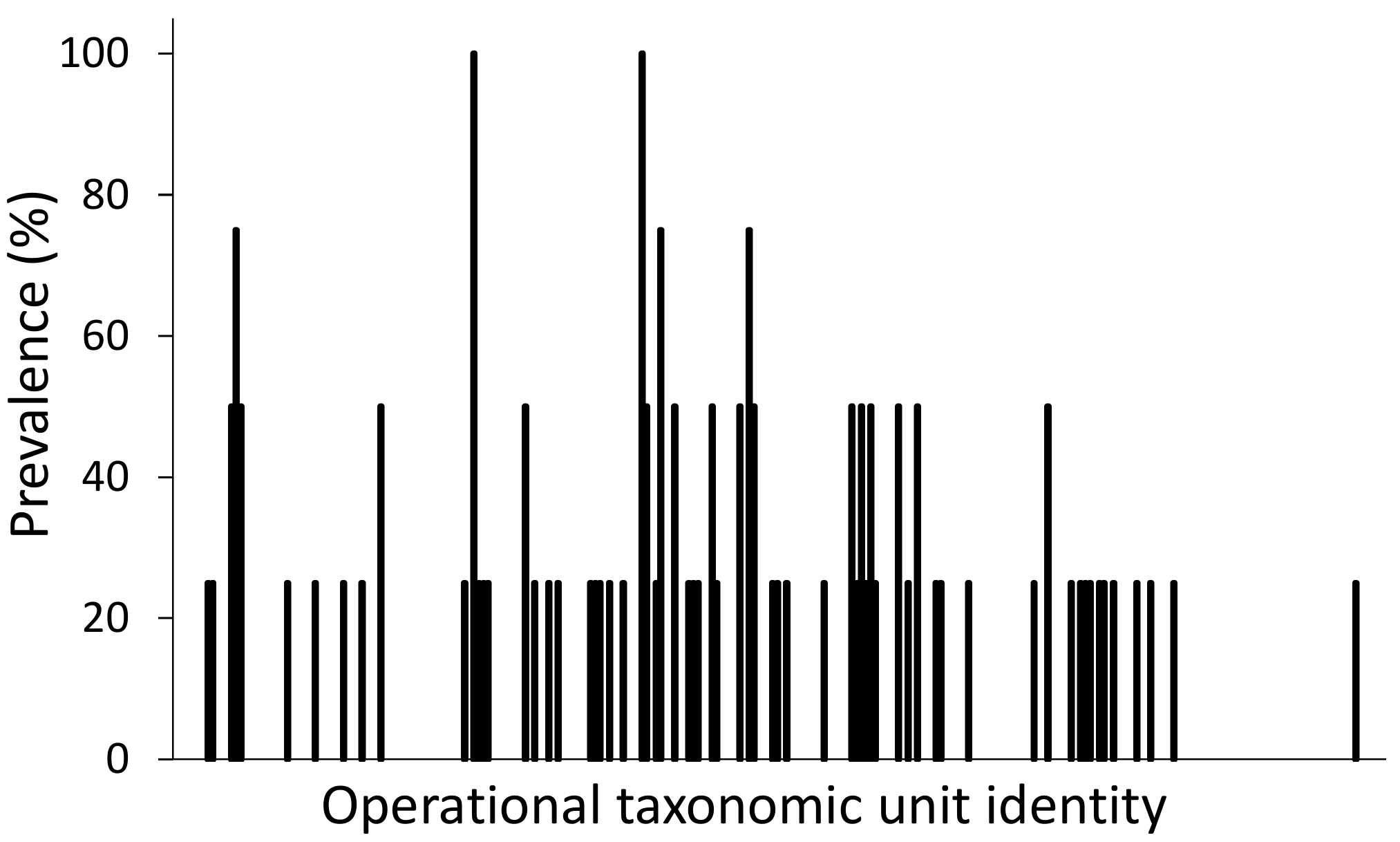
**

**Fig 5h. (little grebe)**

**
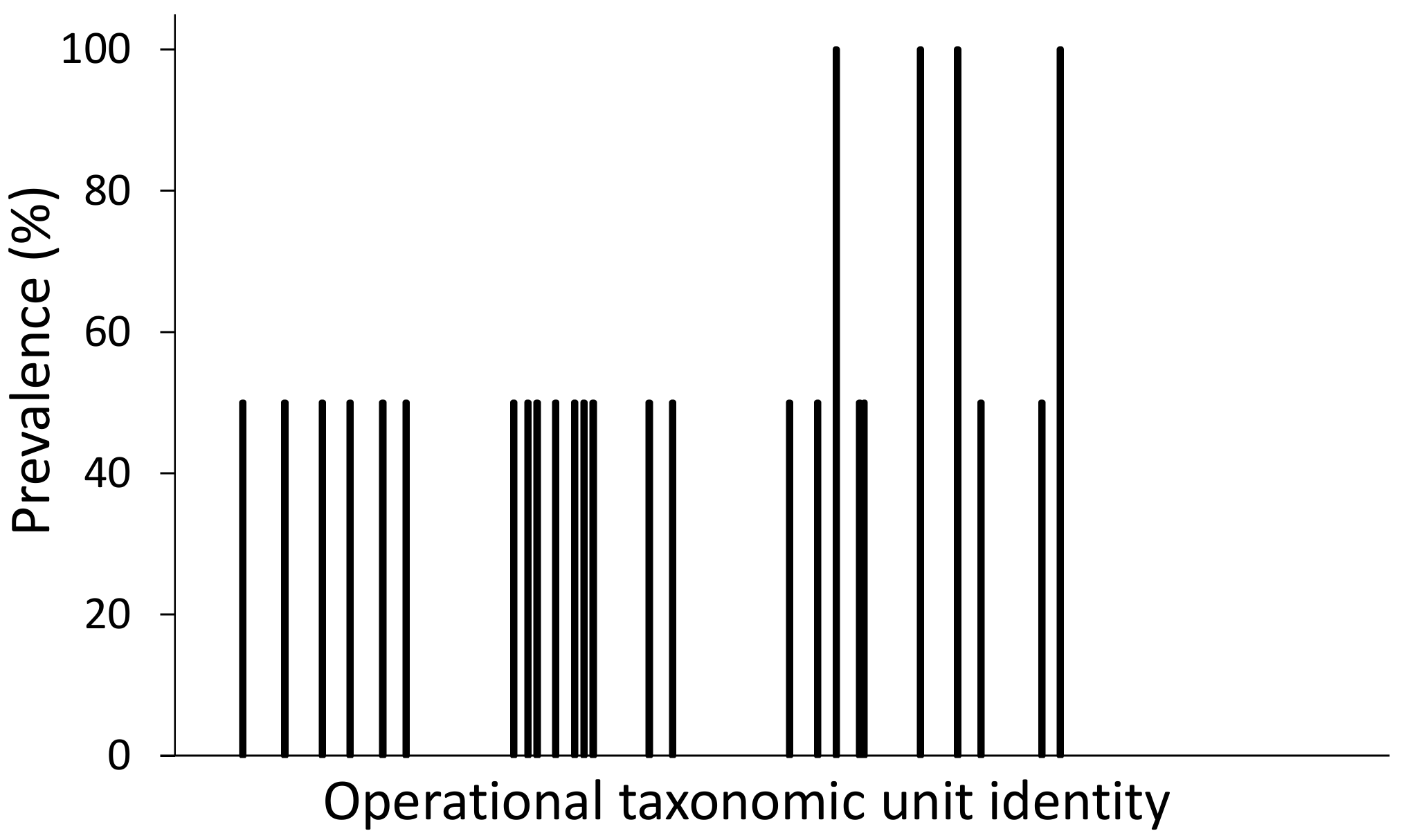
**

**Fig 5i. (mallard)**

**
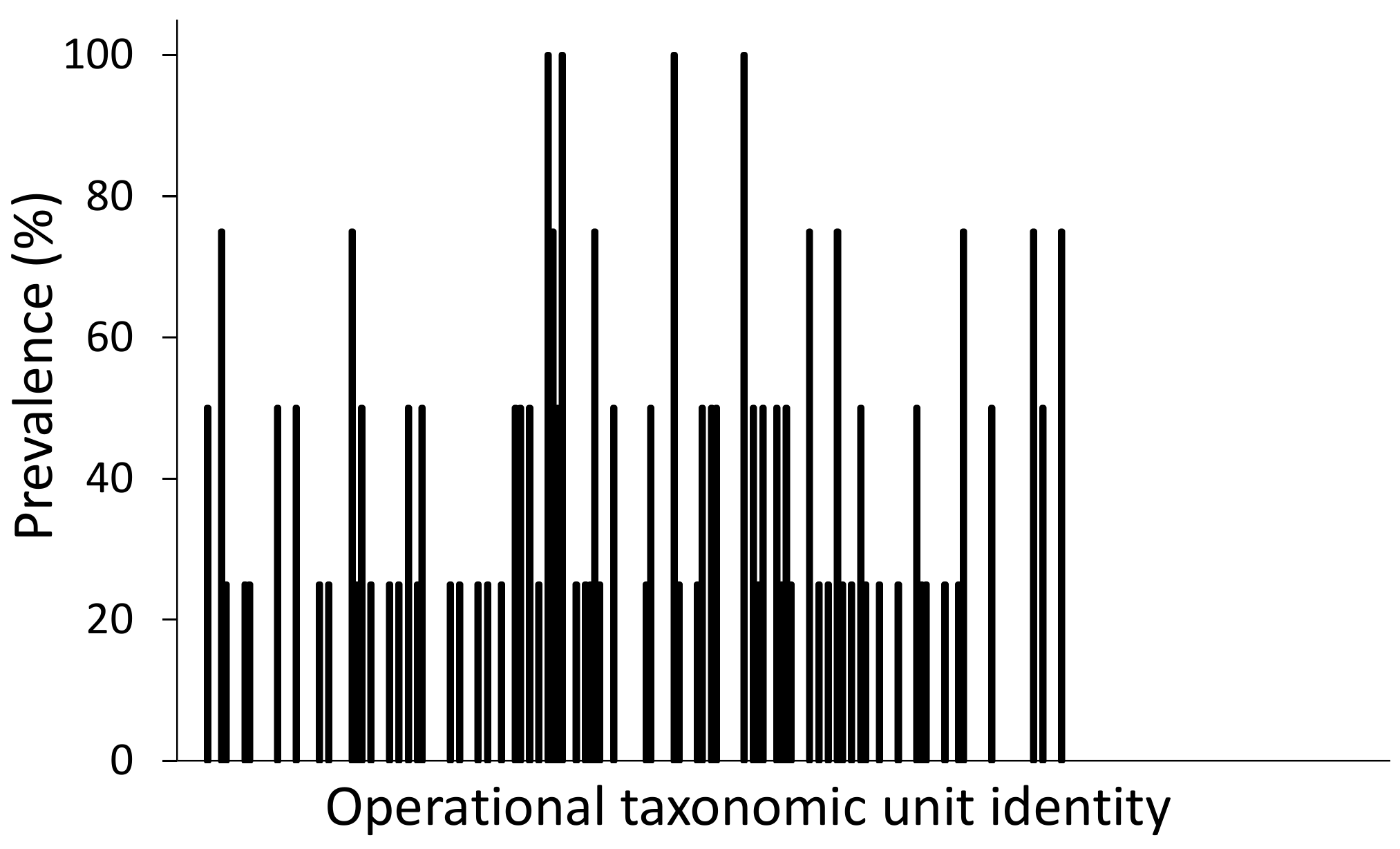
**

**Fig 5j. (mute swan)**

**
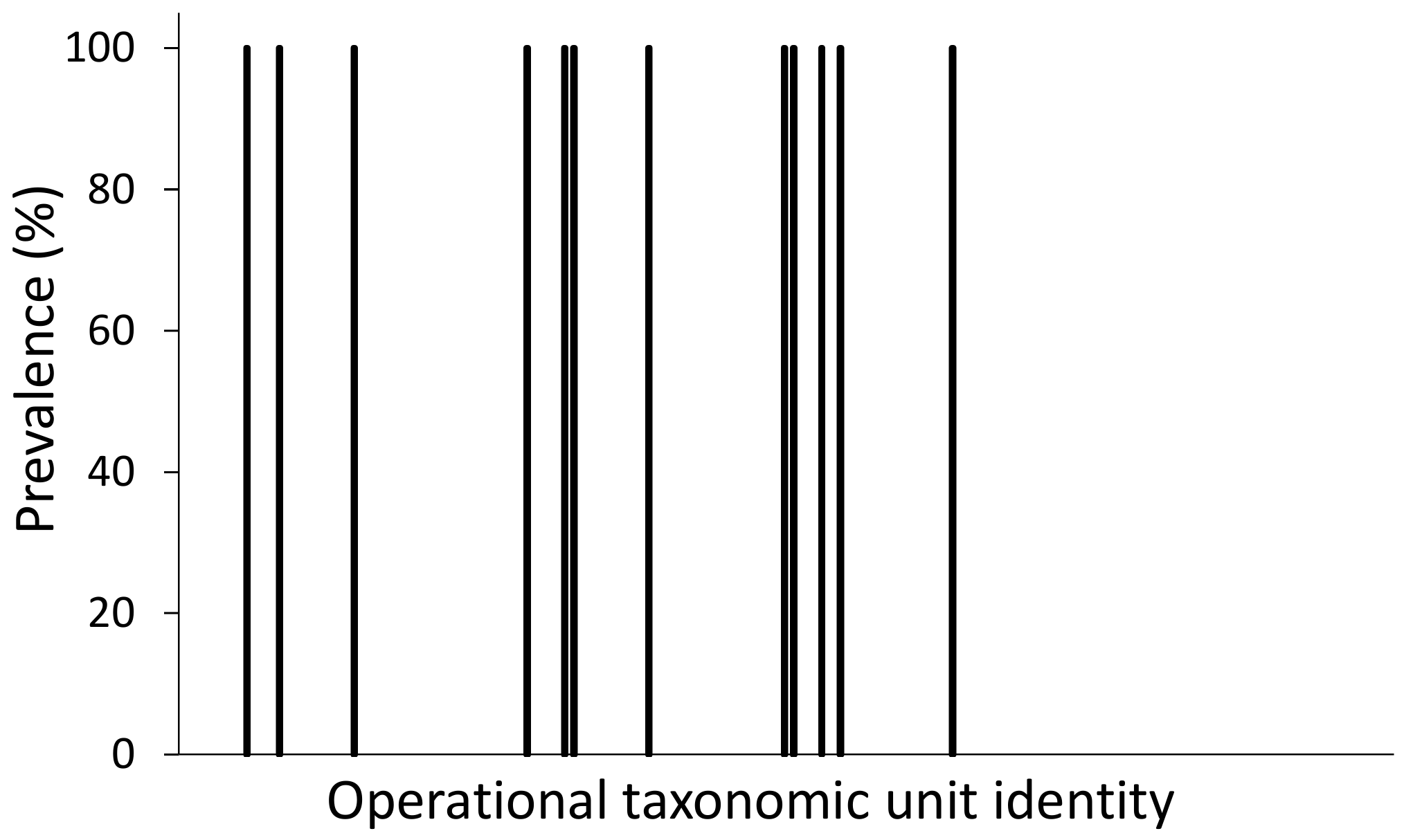
**

**Fig 5k. (purple heron)**

**
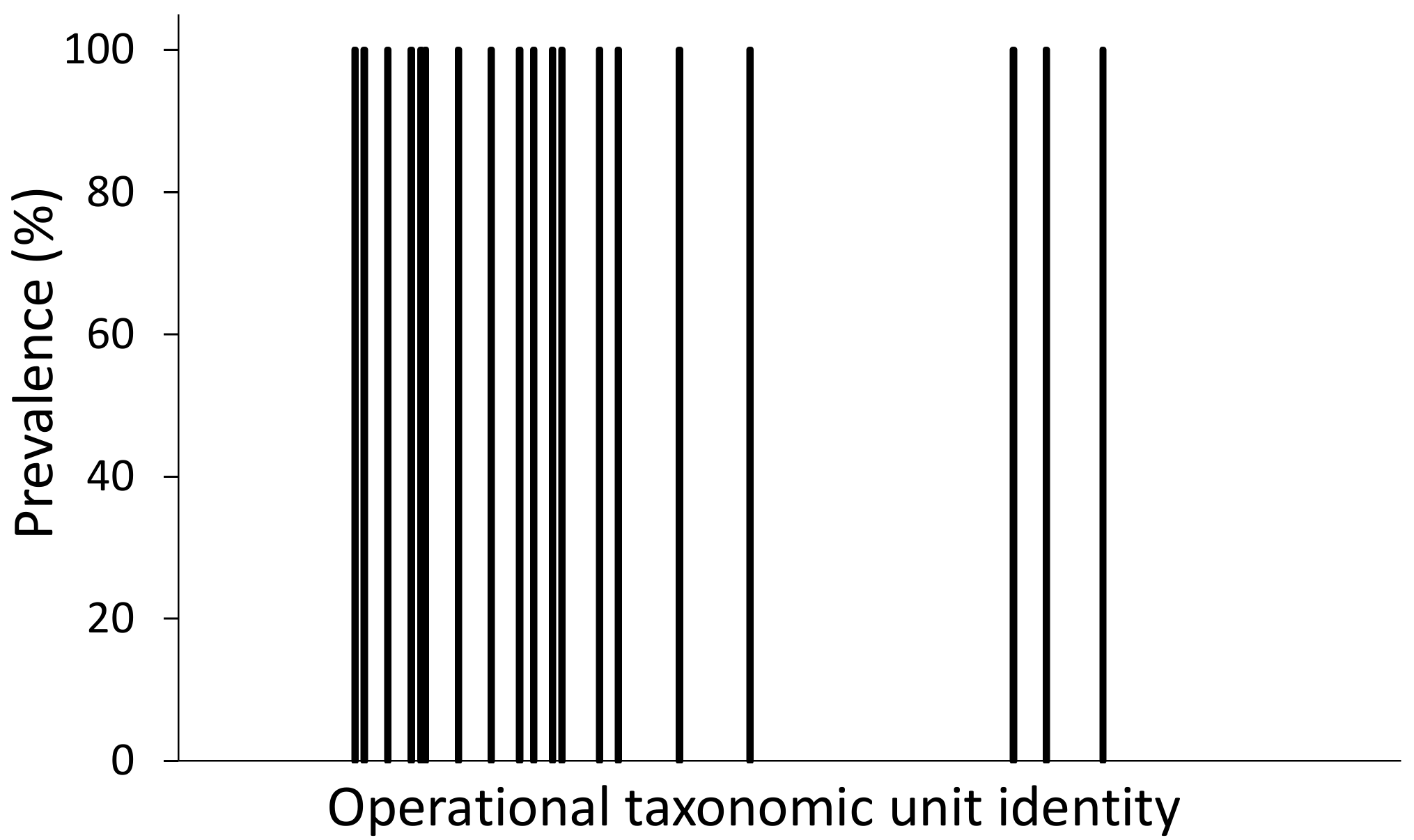
**

**Fig 5l. (red-crested pochard)**

**
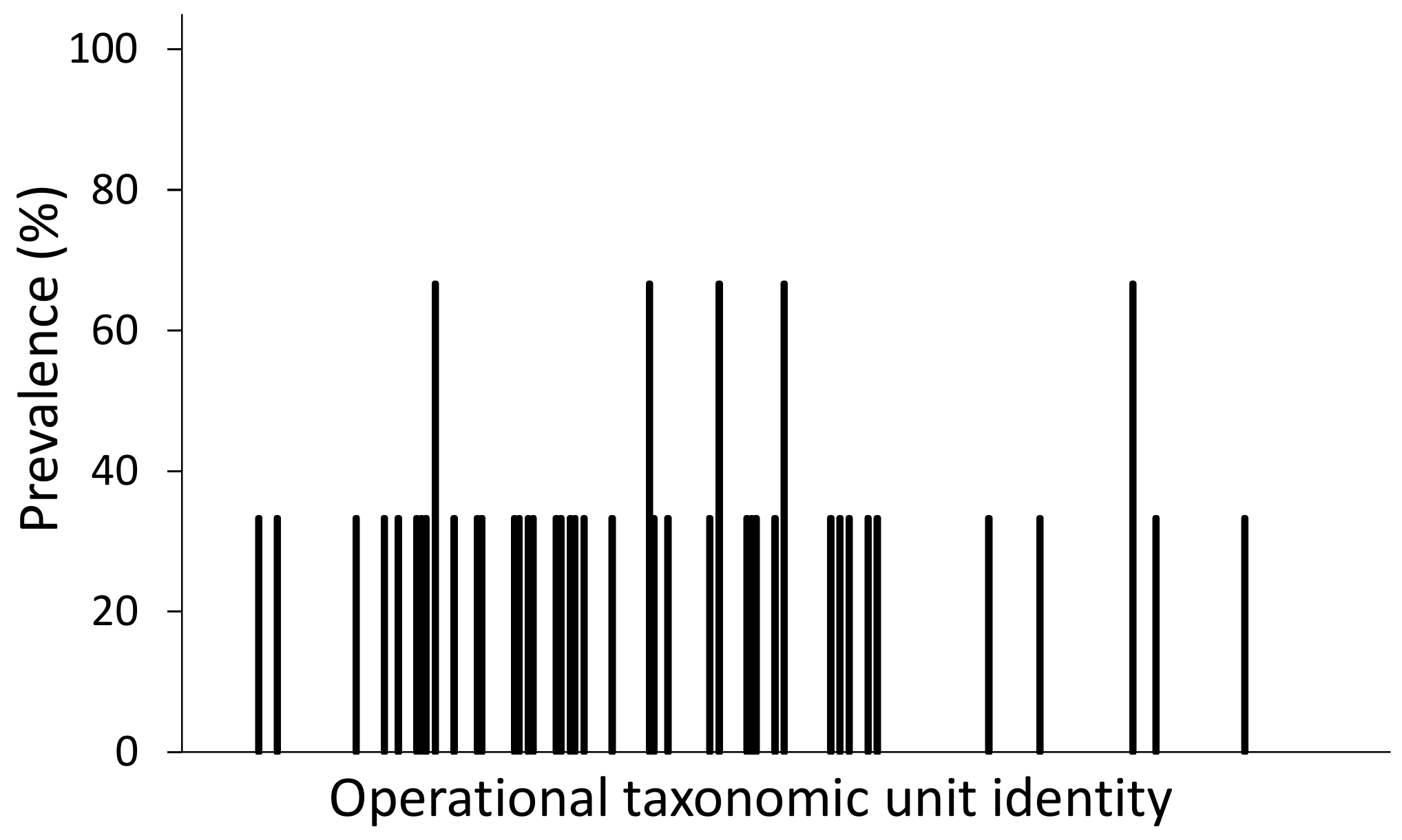
**

**Fig 5m. (Savi’s warbler)**

**
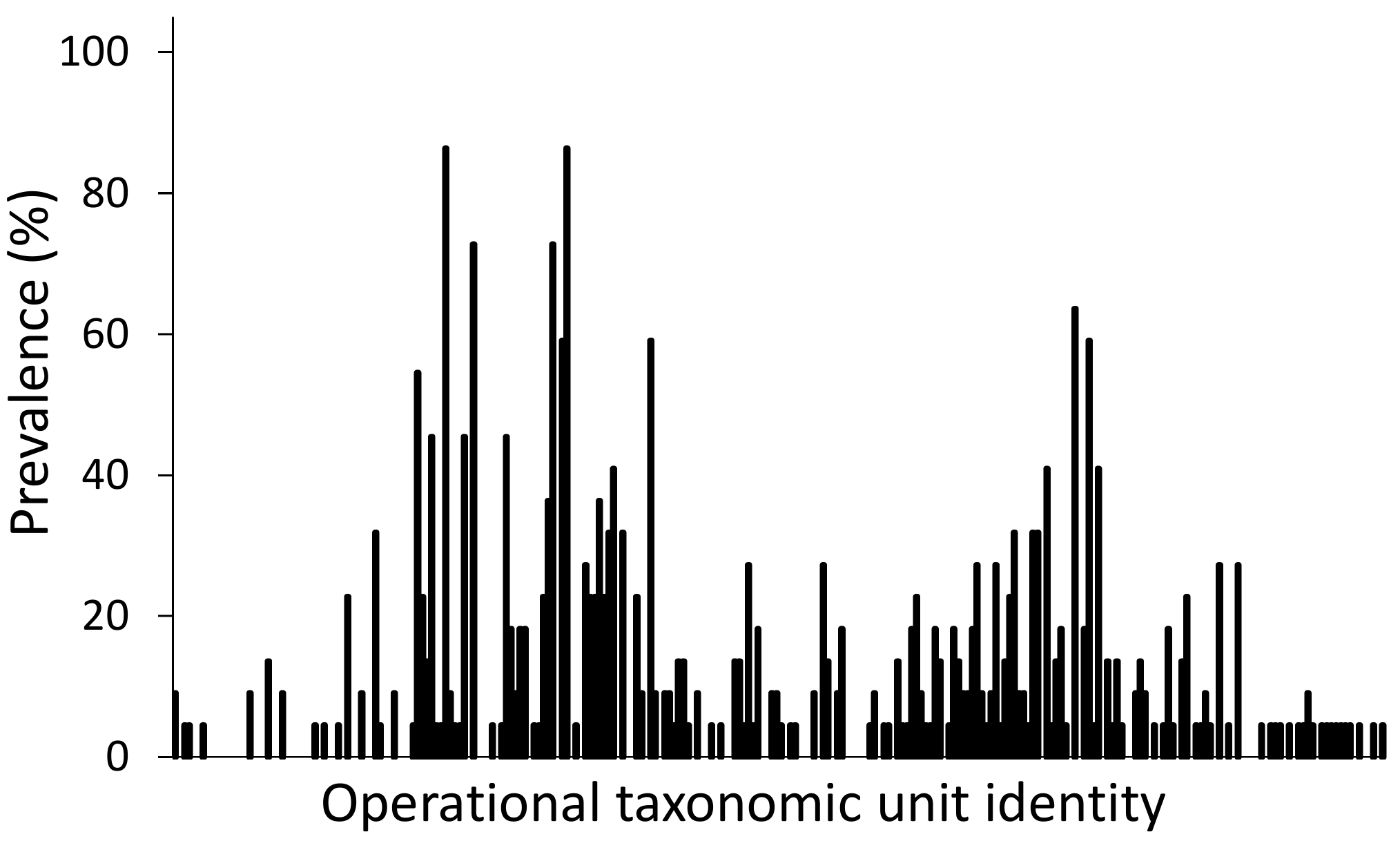
**

**Fig 5n. (water)**
