# Supplementary material for: Bacterial assemblages on eggs reflect nesting strategies in wetland-associated birds
Source: PLoS One. 2025 Sep 17;20(9):e0332380. doi: 10.1371/journal.pone.0332380 (PMC12443268; doi:10.1371/journal.pone.0332380)
Supplement: S6 Fig — Sampling groups are a) water samples, b) common coot egg samples and c) great-crested grebe collected at different locations including Budkovany (black circles), Jakubov (blue circles), Velke Blahovo (white circles) and Vrable (green circles). (DOCX) [file pone.0332380.s006.docx]

**S6 Fig. Non-metric multi-dimensional scaling along the first and second axes outlining differences in bacterial assemblages between sampling groups.** Sampling groups are a) water samples, b) common coot egg samples and c) great crested grebe collected at different locations including Budkovany (black circles), Jakubov (blue circles), Velke Blahovo (white circles) and Vrable (green circles).

**
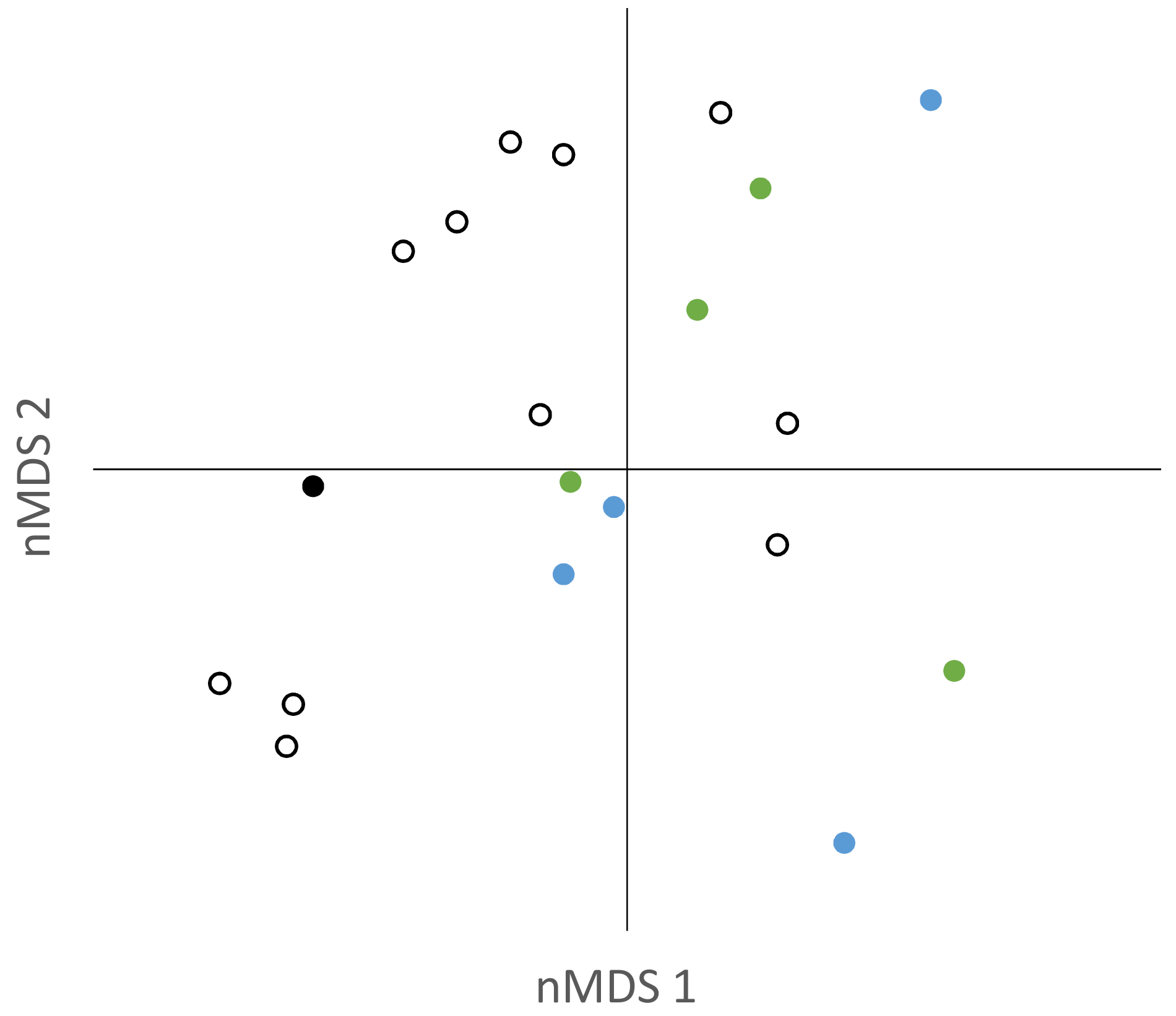
**

**Fig 6a.**

**
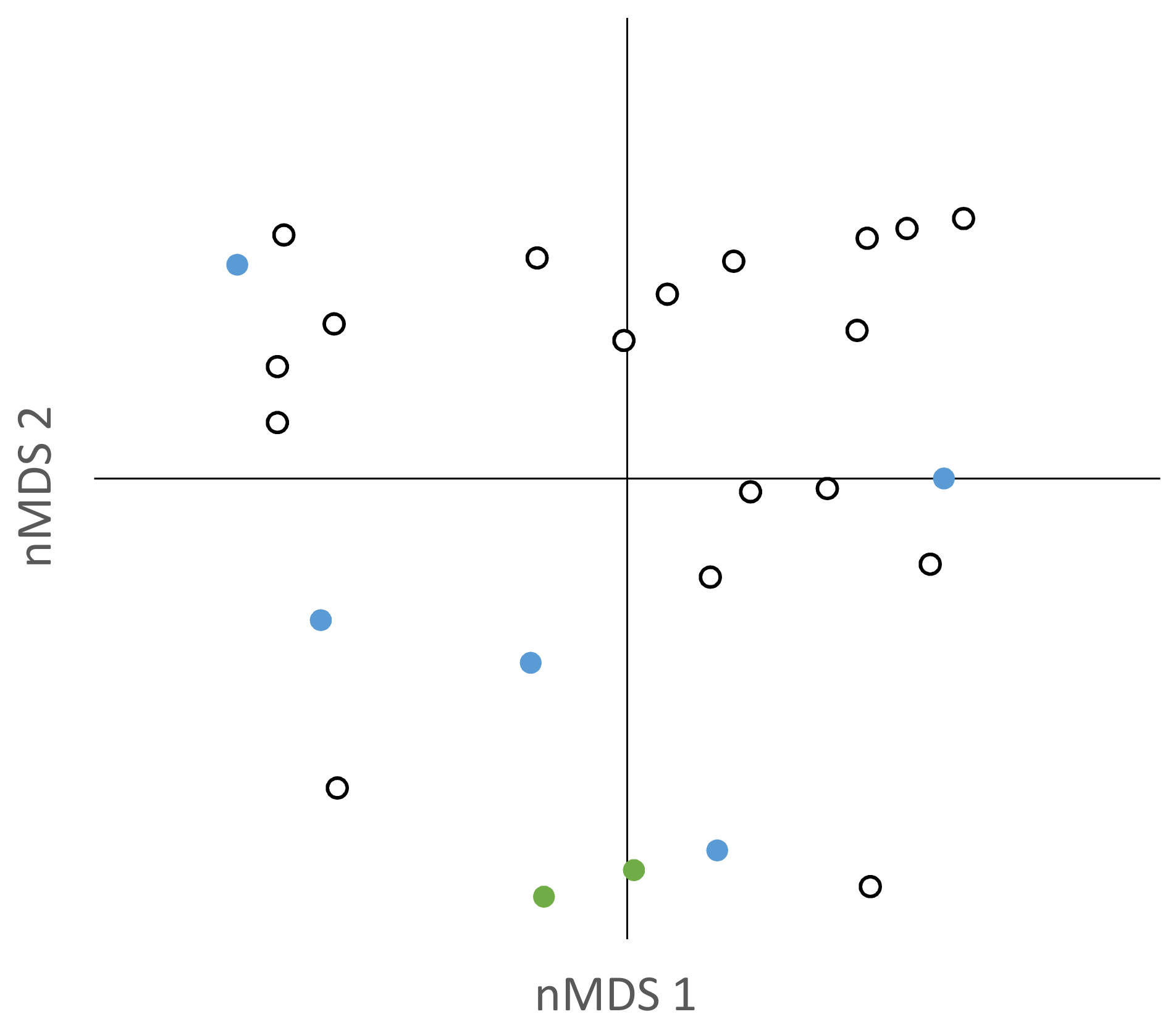
**

**Fig 6b.**

**
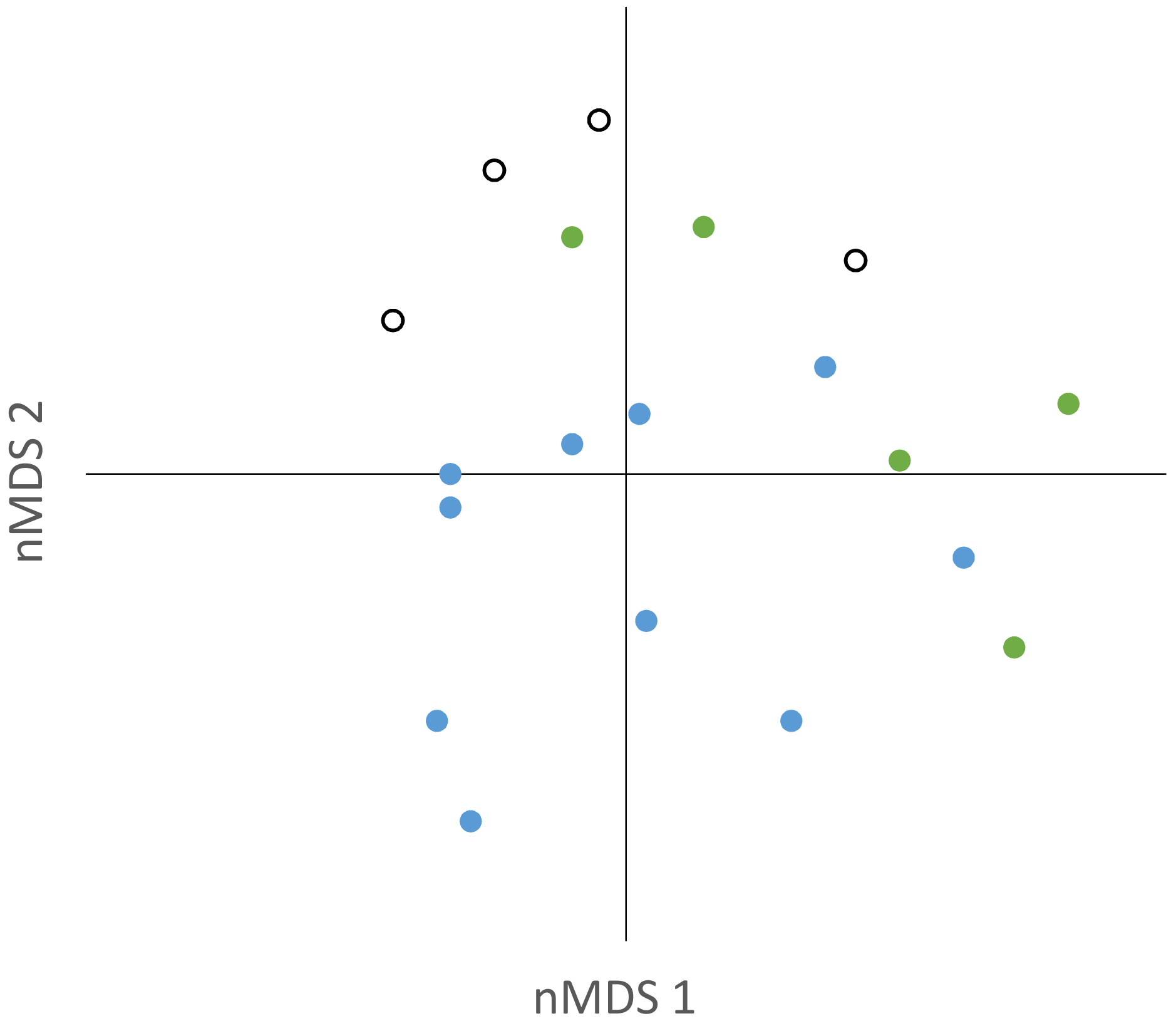
**

**Fig 6c.**
