# Supplementary material for: Bacterial assemblages on eggs reflect nesting strategies in wetland-associated birds
Source: PLoS One. 2025 Sep 17;20(9):e0332380. doi: 10.1371/journal.pone.0332380 (PMC12443268; doi:10.1371/journal.pone.0332380)
Supplement: Attachment — Submitted filename: Review PONE-D-24-57432.pdf [file pone.0332380.s008.pdf]

Review: PONE-D-24-57432 - **Bacterial assemblages on eggs reflect nesting strategies in wetland-associated birds**

Dear Authors. When I read the abstract of your manuscript, I was excited about it because, as you mention, it is the first comparative study looking at the eggshell bacterial assemblage across wetland species with different nesting strategies – wet and dry. However, once I started reading the sampling methodology and statistical testing, my enthusiasm waned slightly. While I find the results regarding the differences in the bacterial community between water samples and on eggs of wet-nesting species mainly interesting supporting the strong nesting microhabitat effect of these wet-nesting strategy species, I have methodological doubts about the study that do not allow for the robust conclusions you state, e.g. that species identity does not play a large role in dry-nesting species. I think this result is more of an artifact of not having a study controlled for the age of the clutch from which you sampled eggshell microbiota from only one egg. Why not from the whole clutch, i.e. each egg, since you didn't control for clutch age and individual eggs at all? I consider this a major shortcoming and either you should add how you checked this, but since you do not mention the nesting stage at which you sampled the eggs at all in the "Material and Methods", rather you did not take this into account at all, you should discuss these limitations and poor sampling within the species sufficiently. I also don't consider it a correct approach to include species in the dry-nesting category that differ significantly in their nesting strategies - e.g. most species nest on the ground, while only a few have elevated nests. Also, ducks deposit their own down-feathers in the nest and also partially incubate the brood, whereas this is no longer typical of other species that represent this dry-nesting group. I suggest a couple of ways to reduce this variability in your data, at least for bacterial load/abundance testing, since you take this into account for ARISA-based OTUs community structure, but not for eggshell bacterial load testing.
Your discussion also needs thorough re-writing– mostly toning down your strong conclusions that are unfortunately based on inappropriate sampling and discussion of your sampling and methodological limitations.

I list my concerns and a possible partial solution below. I hopefully they will help to make your data and outcomes more robust.

Abstract:
Last sentence of abstract sounds a little vague. I understand what you want to express, but better will be use some sentence which stimulate the future research on antimicrobial protection of eggs across habitat humidity gradient or try to be more generalist in your expressing and make some future suggestions.

Material and Methods:

Line 128 – why you did not swab all egg in the nest to have some "common" bacterial assemblage?? Did you candle eggs to assign development stage of each nest-eggs? Difference between first and last egg in the nest may be 7 or more days – especially in the case of ducks. So if you take lastly laid egg (randomly) it can have lower bacterial load compared to first laid egg in the clutch. So, sampling eggshell of only one egg from clutch is really not good sampling approach. Please note, eggshell bacterial assemblage may significantly differ with respect to nesting/incubation period - bacterial assemblages on eggs reflect and vary in fresh

or continuously incubated eggs. Moreover, considering that your sampling design is strongly imbalanced (e.g. 48 Great-crested grebe nests to compared to one Moorhen nest), I see the fatal flaw in your study as you did not control nest/egg age or incubation phase during the sampling period which was quite extensive (May-July). All these factors may strongly affect both, microbial load and assemblage. How you were able to control for this confounding effect in your study. Please justify. Otherwise, your results cannot be considered as reliable with omitting this variability into account and indices of bacterial load and communities may be very biased preventing comparison between individual nests (wet-, dry- breeders).

Line 144 – cultivation of bacteria – you did not run in replicates? How did you count CFU? Manually? Using SW from photography? Be more detailed in description of your technique?

- ARISA- please as this method is not such precise as sequencing, use term ARISA based-OTUs throughout the whole manuscript as term OTUs per se is emerged from more precise sequencing technique than ARISA.

Statistical analyses

To have you sampling design, I first inspect within species variability in bacterial load/abundnace. I am lacking basic exploratory data analysis (min, max, range, mean SD, SE – i.e. expressing variability of data within species. I guess it will be quite extensive. You can do at least for *Fulica atra* and *Podiceps cristatus* where you have nice sampling.

- another factor is covering the egg with the feathers – ducks (Mallards and *Aythya* spp.) use their own body feathers as nest lining material and cover eggs with these feathers. This might be other factor that can strongly affect eggshell microbial assemblage. To see your species and dataset. I am for to extend your statistics from glmer approach to also pairwise comparison of grebes or only *Podiceps cirstatus* with only *Fulica atra* as representative species from wet-, dry -nesting strategy continuum. I see grouping of ducks, Savi warblers and herons as not intuitive with respect to their nest building and nest material specifities. Also, most species are ground nesters but what about heron and Ixobrychus and Savi warbler??? I think you mixed too much together without appropriate controlling of confounding effects.

I am strongly for make more species groups – e.g. put ducks using feathers in the nest together as they really have specific nest building habits via incorporating own body down feathers into the nest and cover the eggs with them. You can do this statistic as additional testing that can uncover another specifities in eggshell bacterial load. Now I see you did for bacterial communities' statistics, but why you did not do also for bacterial load (abundance) data??? E.g in Figure 1 I like to see boxplots where wet-nesters will be depictes separately, e.g. ducks also separately, and other species as well – except of these having n=1). I think it will be more reliable than to mix all dry-nesters together into one group consisting of many species with distinct nesting strategy.

Discussion

Line 316-320 – Sorry but this conclusion is too strong considering your sampling effort one to three nest per most of the dry-nesting species. Based on your dataset and fatal flaw with ignoring incubation phase in sampling egg, you cannot rule out the role of species identity on

eggshell bacterial assemblage. Tone down this your conclusions and rather discuss thoroughly possible limitations of your study that more robust sampling controlled for nesting phase of sampling eggs in necessary to test these assumptions – i.e. rule out or test the effect of species identity on eggshell bacterial load. and discuss all these shortcomings of your study.

Lines 327-329 – also this conclusion needs to additional testing as I suggested. Use only dry-nesting species where appropriate number of individuals you have or cluster together ducks that have very specific nesting – incorporating of own body down feathers into the nest lining, egg covering and partial incubation..I think it will help to dilute all confounding effects that were omitted in your comparison and clustering all dry-nesting species in one group…

Lines 336 –337 - of course if you sample one to three nest per species and sample only one egg per clutch. It is not representative eggshell microbial assemblage per species, sorry, but you need more robust sampling to see some nesting ecology-based clustering.

Line 338 – yes, and also cover their eggs with nesting material full of down feathers that may have thermoregulatory and also antimicrobial properties. Also intermitently incubate their eggs before starting full incubating which affect both, eggshell, but also egg white bacterial load – see e.g. Effect of intermittent incubation and clutch covering on the probability of bacterial trans-shell infection | Semantic Scholar, or Laying date, incubation and egg breakage as determinants of bacterial load on bird eggshells: experimental evidence | Oecologia
You should take into account also this very specific nesting ecology before choosing species for your study…or at least take this into account during testing your hypotheses and discuss these specifications thoroughly.

Lines 343-344 and further – tone down thoroughly.. You really don´t have data on these strong conclusions.

Lines 346-348 – and also more thorough sampling of dry-nesting wetland species…

Lines 365-376 – but there is also studies that did not find and effect and in general there is no study testing direct effect of trans-shell penetration and embryo mortality. Discuss more precisely. It is not such straightforward as you described. Most of eggshell bacterial are not able to live inside the eggs etc. etc…

Line 357 – yes, incubation, and how did you control this in your study??? Try to put this infor into the context of your study. It is not discussion, just plain informative manner which you used here..

Line 366 – your findings may highlight something but are not such robust, unfortunately..So try to tone down that your findings may serve as some initial stimuli for further more thorough testing…
