# Supplementary material for: Bacterial assemblages on eggs reflect nesting strategies in wetland-associated birds
Source: PLoS One. 2025 Sep 17;20(9):e0332380. doi: 10.1371/journal.pone.0332380 (PMC12443268; doi:10.1371/journal.pone.0332380)
Supplement: Attachment — Submitted filename: Review PONE-D-24-57432_R1.pdf [file pone.0332380.s010.pdf]

Review: PONE-D-24-57432_R1 - **Bacterial assemblages on eggs reflect nesting strategies in wetland-associated birds**

Dear authors. I am fully satisfied with the revised version of your manuscript. I think you improved the clarity and comprehensively toned down your conclusions and now the study is really useful as a stimuli for futures studies focused on eggshell bacterial assemblages and/or bacterial trans-shell penetration in the context of breeding ecology of birds.

I have only a few minor comments and suggestions which you can find below:

Technical comment:

Why you changed corresponding author in the revised version of the manuscript and why one of the co-authors (L. Rubáčová) has been removed from the author team to Acknowledgements only? Did she agree with this and it was discussed with her thoroughly? It is not usual such dramatically change author membership and status of authors?

To your comments regarding clutch age (my (Reviewer 3) Comment 1) and effect on eggshell bacterial assemblage. Yes, you considered sampling date and describe that effect might be linked with changed environmental factors. But, I am considering incubation of the clutch and resulting eggshell assemblage. As you described on lines 150-152, you sampled one egg from nest when you found it, but although you record the precise date of nest finding, you were not aware of the age of the clutch (i.e. how long the clutch has been incubated already). Moreover, it is quite common that 1) nesting parasitism occurred in Mallards and also coot and grebes and also that 2) intermittent incubation of partial not-completed clutch occurred before the full incubation and clutch completion (at least in ducks it is very common, and I think also swans or grebes are known for this behaviour). In practice, simple fieldwork "candler" (inspection of egg inner structure under the light) is used for determination of embryo age which correspond to intensity of incubation. And as you mentioned in you text of the revised ms that incubation may affect eggshell bacterial assemblage (and quite a lot), this discrepancy in eggs age and developmental stage within the clutch may cause bias if your chosen only one egg from the complete clutch for sampling. I think you should somehow touch of this possible bias in your data caused by the different clutch age, role of intermittent incubation and/or brood parasitism in your focal species.

But in general, it is nice you added additional GLMMs testing for the effect of clutch size and sampling date, as it is really highly probably that enlarged clutches may be incubated before clutch completion or being parasitized by brood parasites. This additional GLMMs made your conclusions more robust. Yet, still, as your study is pioneering in study of bacteria on eggshell with respect to breeding ecology, I am for to include possible factors that might be behind the differences between dry- and wet-nesters. Although I understand your point of view and you want to keep the line of the study as simple as possible, I thin a few sentences in the discussion with other potential factors cannot be distracting from the major message of your study.

Line 141 – "more holistic overview"., I suggest to replace holistic by "comprehensive". Holistic is really to strong meaning in your case study.

Line 142 - …in isolation. I suggest to replace by "separately" or some other equivalent.

Line 290 – "….of bacteria on eggshells on wet-nesting.." rephrase it please.

Line 295 – "coliforms" without capital please

Lines 359-366 – I am still wondering if it cannot be partially caused by pre-incubation behaviour of dry-nesters – covering of clutch by down feathers in mallard and other duck species, intermittent incubation I have mentioned above..I think you should take this into account in discussion of your results (mostly differences between dry and wet-nesters). You already did for covering the clutch by feathers, and even if you disagree to do for incubation (see response to my Comments 18). I think it can help to untangle the all possible confounding factors that have to be taken into account in future studies of eggshell or trans-shell bacterial assemblages.

Line 380 The aim of this study…..I suggest to rephrase… "…bacterial abundance and community structure vary based on……".

Your Response to Comment 17 of Reviewer 3 – for example this study has show no effect of bacterial trans-shell penetration on hatching success: Effect of intermittent incubation and clutch covering on the probability of bacterial trans-shell infection - Javůrková - 2014 - Ibis - Wiley Online Library
