# Supplementary material for: Bacterial assemblages on eggs reflect nesting strategies in wetland-associated birds
Source: PLoS One. 2025 Sep 17;20(9):e0332380. doi: 10.1371/journal.pone.0332380 (PMC12443268; doi:10.1371/journal.pone.0332380)
Supplement: Attachment — Submitted filename: Response to Reviewers.docx [file pone.0332380.s011.docx]

**Reviewer 1**

**Comment 1:** *In future studies, it is recommended to increase the sample size and expand the number of samples for dry-nesting species to more accurately assess the impact of nesting strategies. Enhancing data reliability by increasing the number of dry-nesting species samples is crucial.*

**Response:** We agree with this comment, and comments made by other reviewers with respect to the relatively low sample sizes in our study. We acknowledge this limitation in both the Results and Discussion. In the Results, we state that our lower sample sizes limit the analyses we can complete (e.g. controlling for covariables such as sampling date, clutch size etc; lines 201-202). In the Discussion, we acknowledge that larger sample sizes are needed to validate our results (both an increase in wet-nesting species, and the number of individuals sampled per species. Lines 374 and 406-413). However, we also highlight that despite these lower sample sizes we are still able to demonstrate novel differences in bacterial abundance and diversity with nesting strategy in wetland associated birds, which is the first study to do so. Our findings provide valuable insights into the challenges associated with nesting in humid environments and it is hoped our research will stimulate further research into the consequences and adaptations of birds nesting in humid environments.

**Comment 2:** *The authors independently reported results from both culture-independent (direct DNA extraction and ARISA analysis) and culture-dependent (bacterial culturing) methods. However, by detailing the relationship and differences between the bacterial communities detected via ARISA analysis and the culturing results, the complementary roles of both methods can be clarified. In addition, it would be beneficial to discuss the extent to which the culture-independent method captures bacterial diversity compared to the culture-dependent method, and whether there are any biases towards specific bacterial communities.*

**Response:** We agree and now add a discussion in the Methods comparing the advantages and limitations of culture-based and ARISA techniques (lines 121-138). We argue that both techniques have their limitations, but together provide a more holistic overview of bacterial loads and diversity. We are not aware of any study that has shown that ARISA displays a bias towards specific bacterial taxa. We are happy to add such a reference should the reviewer be aware of one.

**Comment 3:** *The introduction explains vertical transmission of eggshell bacteria in birds (transmission from the mother within the oviduct before eggshell formation). However, this study does not clearly distinguish whether the eggshell bacteria are derived from maternal contact or from other environmental sources. A discussion including comparisons with the bacterial communities present on the exterior and interior of the mother bird is necessary. Please discuss comparative analyses between the mother's bacterial communities (such as those in the cloaca or skin microbiota) and those on the eggshell to identify maternally transmitted bacteria.*

**Response:** The mechanisms determining the acquisition of eggshell bacteria are of central importance to this study, although our data do not allow a distinction between environmentally- and maternally-acquired bacteria. We acknowledge that these two potential bacterial sources need to be mentioned, which we now do in the opening paragraph of the Discussion (lines 358-364). As our data do not permit a deeper exploration of horizontal vs vertical transmission (we did not swab maternal skin or cloaca), we do not provide further discussion on how different bacterial strains may be acquired via the two vectors.

**Comment 4:** *The terms "wet-nesting" and "dry-nesting" are inconsistently hyphenated throughout the manuscript. It is recommended to standardize the hyphenation for consistency.*

**Response:** Thank you. We have now gone through the manuscript and hyphenated all occurrences of the terms.

**Reviewer 2**
**Comment 1:** *The authors determine bacterial abundance by selective and non-selective plating on bacterial growth plates. While this is fine for certain bacterial taxa, there is a risk of the isolated colonies not being representative of the actual microbial community, only the microbes capable of being cultured on agar. Performing 16S qPCR would be more accurately indicative of true bacterial abundance.*

**Response:** We acknowledge the inherent limitations of culture-based methods and now discuss this in more detail in lines 121-138. We discuss the limitations of both culture-based techniques and ARISA, and that more advanced techniques exist. We also argue that, when used together, our methods still represent an informative tool in microbiology when focusing on broad differences in bacterial assemblages between treatment groups. Future studies would indeed benefit from using more modern techniques, such as 16S qPCR.

**Comment 2:** *The authors use automated ribosomal intergenic space analysis (ARISA) to detect and report OTUs from their sampled nests. While an established assay, ARISA sports few benefits over newer, more powerful techniques such as 16S sequencing or shotgun metagenomics. These techniques would allow the authors to better characterize the exact differences between the microbial communities under research.*

**Response:** We agree that the methods we have used here are now infrequently used due to advances in molecular techniques. We have acknowledged the inherent limitations of culture-based and ARISA techniques, but argue that despite these limitations, they remain an effective tool in characterising broad differences in bacterial communities between treatment groups. Indeed, we here have successfully used these techniques here to demonstrate that nesting strategy can influence eggshell bacterial communities in wetland-associated birds. We however recognize the potential benefit of integrating 16S qPCR in future studies to further validate our findings. We discuss these limitations in lines 121-138.

**Comment 3:** *Line 144 – the word “media” is missing.*

**Response:** ‘Media’ added

**Comment 4:** *Figure 1 – it would be helpful to include a legend in the figure itself indicating that white bars are dry-nesting birds and black bars are wet-nesting birds.*

**Response:** We prefer not to add a key, as this would add to cluttering within the figure. We have therefore left the explanation of the white and black bars in the caption (which will be located directly below the figure in the final publication).

**Comment 5:** *Figure 1 – Statistical significance would be helpful to report in the figure itself using the * system for noting p-values.*

**Response:** We have now added asterisks in the figure to denote significance levels.

**Comment 6:** *Figure 2 – Authors should note in the text the differences between figures 2a, 2b, and 2c.*

**Response:** We apologise, but we currently do not understand what the reviewer is requesting here. The three figures simply show the same MDS data along the three different axes. If the reviewer could clarify their suggestion, we would be happy to respond.

**Reviewer 3**
**Comment 1:** *I have methodological doubts about the study that do not allow for the robust conclusions you state, e.g. that species identity does not play a large role in dry-nesting species. I think this result is more of an artifact of not having a study controlled for the age of the clutch from which you sampled eggshell microbiota from only one egg. Why not from the whole clutch, i.e. each egg, since you didn't control for clutch age and individual eggs at all? I consider this a major shortcoming and either you should add how you checked this, but since you do not mention the nesting stage at which you sampled the eggs at all in the "Material and Methods", rather you did not take this into account at all, you should discuss these limitations and poor sampling within the species sufficiently.*

**Response:** After careful consideration, we agree with the reviewer that our analyses are not robust enough to determine if species identity play an important role in determining bacterial loads, given the low sample sizes for most species. We have therefore removed all previous discussion that nesting-strategy was a more important determinant of bacterial assemblage than species identity (throughout both the Abstract and Discussion). We also acknowledge the limitations associated with the low number of wet-nesting species in our study and the low number of individuals for which we have samples for dry-nesting species (lines 201, 374 and 406-413). Despite these limitations, we argue that we can still demonstrate the impact of nesting strategy on bacterial assemblages, which was the primary objective of this study.

We are unsure how our findings may be an artifact of not controlling for clutch age, as suggested by the reviewer. We are therefore happy to receive further feedback on this. However, we now show in the common coot and great-crested grebe, that the number of eggs in the nest and sampling date generally do not affect bacterial loads (except for in Coliforms, where bacterial abundance increases with sampling date; lines 213-219, 282-285 and S3 Table). This suggests that clutch age may not have a strong effect on eggshell bacterial communities, although further study is required to verify this.

With regards to our decision to only sample one egg per clutch, this was to maintain consistent methodology across species and to maximise the number of nests we could sample. The mean number of eggs per nest are highly variable between species, including great-crested grebe (3.8 eggs/nest), little grebe (5.4 eggs/nest), mute swan (5 .6 eggs/nest), common coot (7.4 eggs/nest) and mallard (9.2 eggs/nest). Sampling all eggs in all nests would result in significant variation in sample sizes between species and limit the number of nests we could sample due to resource constraints. We instead prioritised sampling a higher number of nests, rather than fewer nests but with more eggs. Finally, previous research has demonstrated consistency in egg-shell bacteria between, and hence presumably within, nests (e.g. Campos-Cerda, F., Torres, R., Nava, L. et al. 2023. Eggshell microbiome as a potential microbial reservoir in a cavity nesting bird. J Ornithol 164, 217–222). This latter result again suggests it would be more worthwhile sampling nests from a greater number of species, rather than more eggs within a single species.

**Comment 2:** *I also don't consider it a correct approach to include species in the dry-nesting category that differ significantly in their nesting strategies - e.g. most species nest on the ground, while only a few have elevated nests. Also, ducks deposit their own down-feathers in the nest and also partially incubate the brood, whereas this is no longer typical of other species that represent this dry-nesting group. I suggest a couple of ways to reduce this variability in your data, at least for bacterial load/abundance testing, since you take this into account for ARISA-based OTUs community structure, but not for eggshell bacterial load testing.*

**Response:** See our response below (Reviewer 2, Comment 10).

**Comment 3:** *Your discussion also needs thorough re-writing– mostly toning down your strong conclusions that are unfortunately based on inappropriate sampling and discussion of your sampling and methodological limitations.*

**Response:** As discussed throughout this reply to the reviewers, we agree that, based on our methodology, our conclusions were too strong in the original version of this manuscript. We have either toned down our conclusions, removed statements that are not supported by the data or added additional discussion to clarify our results. For example, we no longer include our earlier statements that species identity does not influence bacterial community (e.g. in the Abstract, and in the opening and concluding lines in Discussions) and acknowledge that larger sample sizes are required to validate our findings (lines 374 and 406-413).

Abstract:
**Comment 4:** *Last sentence of abstract sounds a little vague. I understand what you want to express, but better will be use some sentence which stimulate the future research on antimicrobial protection of eggs across habitat humidity gradient or try to be more generalist in your expressing and make some future suggestions.*

**Response:** We agree and now state: “Our research suggests that nesting in moist conditions presents unique challenges to wetland birds and highlights the potential for future research into the unique antimicrobial adaptations associated with the eggshells of these species” (lines 39-42).

Material and Methods:
**Comment 5:** Line 143 – why you did not swab all egg in the nest to have some “common” bacterial assemblage??

**Response:** Please see our response to Reviewer 3, Comment 1 (final paragraph).

**Comment 6:** *Did you candle eggs to assign development stage of each nest-eggs? Difference between first and last egg in the nest may be 7 or more days – especially in the case of ducks. So if you take lastly laid egg (randomly) it can have lower bacterial load compared to first laid egg in the clutch. So, sampling eggshell of only one egg from clutch is really not good sampling approach. Please note, eggshell bacterial assemblage may significantly differ with respect to nesting/incubation period - bacterial assemblages on eggs reflect and vary in fresh or continuously incubated eggs. Moreover, considering that your sampling design is strongly imbalanced (e.g. 48 Great-crested grebe nests to compared to one Moorhen nest), I see the fatal flaw in your study as you did not control nest/egg age or incubation phase during the sampling period which was quite extensive (May-July). All these factors may strongly affect both, microbial load and assemblage. How you were able to control for this confounding effect in your study. Please justify. Otherwise, your results cannot be considered as reliable with omitting this variability into account and indices of bacterial load and communities may be very biased preventing comparison between individual nests (wet-, dry- breeders).*

**Response:** We acknowledge that eggshell bacteria are likely to vary with egg age due to various factors, such as incubation. We also acknowledge that this temporal variation will likely add additional variability in eggshell assemblages that we have not quantified, although the exact day that incubation begins can be difficult to determine due to partial incubation (Wang and Beissinger 2011), which is now mentioned in lines 211-212. What we find fascinating is that we find clustering in bacterial assemblages between wet- and dry-nesters DESPITE these additional sources of variation. Indeed, due to our relatively low sample sizes (see discussion elsewhere in our response to reviewers), including additional covariables in our models (such as egg age) would likely lead to overfitting and potentially spurious results (see lines 201-219 for a discussion of this). Finally, our additional analyses on the great-crested grebe and the common coot, for which we had larger sample sizes, show minimal to no impact of the number of eggs (and thus the likelihood of incubation) or sampling date on bacterial communities (lines 282-285 and S3 Table). This further supports our decision to exclude additional covariables from our analyses. Future studies would benefit from collecting data from a larger number of nests and focusing on temporal variation in subsequent statistical analyses.

Jennifer M. Wang, Steven R. Beissinger, Partial Incubation in Birds: Its Occurrence, Function, and Quantification, *The Auk*, Volume 128, Issue 3, 1 July 2011, Pages 454–466,

**Comment 7:** *Line 161 – cultivation of bacteria – you did not run in replicates? How did you count CFU? Manually? Using SW from photography? Be more detailed in description of your technique?*

**Response:** We now state that “CFU abundance was counted directly from plates when CFU density was low. We took a photograph of high-density plates, magnified the image on a screen and marked each CFU as it was counted” (lines 157-159). We did not run replicates, but are confident that our estimates of abundance were accurate, due to the known repeatability of culture-based quantification from previous studies (e.g. Jarvis et al, 2007).

Jarvis, B., Hedges, A. J., & Corry, J. E. (2007). Assessment of measurement uncertainty for quantitative methods of analysis: comparative assessment of the precision (uncertainty) of bacterial colony counts. International journal of food microbiology, 116(1), 44–51.

**Comment 8:** *ARISA- please as this method is not such precise as sequencing, use term ARISA based-OTUs throughout the whole manuscript as term OTUs per se is emerged from more precise sequencing technique than ARISA.*

**Response:** We agree that OTUs is now associated with a more precise sequencing technique. However, we feel that always referring to OTUs as ARISA-OTU in this manuscript would clutter the text (twenty-four mentions through-out text, often multiple times per sentence) and that it is clear from the methods that we are referring to ARISA OTUs. We therefore prefer to keep the text as is.

Statistical analyses
**Comment 9:** *To have you sampling design, I first inspect within species variability in bacterial load/abundance. I am lacking basic exploratory data analysis (min, max, range, mean SD, SE – i.e. expressing variability of data within species. I guess it will be quite extensive. You can do at least for Fulica atra and Podiceps cristatus where you have nice sampling.*

**Response:** Thank you for this suggestion. We have now added an additional table in the Supplementary material with cultured bacteria descriptive statistics for all study species (S2 Table).

**Comment 10:** *Another factor is covering the egg with the feathers – ducks (Mallards and Aythya spp.) use their own body feathers as nest lining material and cover eggs with these feathers. This might be other factor that can strongly affect eggshell microbial assemblage. To see your species and dataset. I am for to extend your statistics from glmer approach to also pairwise comparison of grebes or only Podiceps cirstatus with only Fulica atra as representative species from wet-, dry -nesting strategy continuum. I see grouping of ducks, Savi warblers and herons as not intuitive with respect to their nest building and nest material specifities. Also, most species are ground nesters but what about heron and Ixobrychus and Savi warbler??? I think you mixed too much together without appropriate controlling of confounding effects. I am strongly for make more species groups – e.g. put ducks using feathers in the nest together as they really have specific nest building habits via incorporating own body down feathers into the nest and cover the eggs with them. You can do this statistic as additional testing that can uncover another specifities in eggshell bacterial load. Now I see you did for bacterial communities’ statistics, but why you did not do also for bacterial load (abundance) data??? E.g in Figure 1 I like to see boxplots where wet-nesters will be depicted separately, e.g. ducks also separately, and other species as well – except of these having n=1). I think it will be more reliable than to mix all dry-nesters together into one group consisting of many species with distinct nesting strategy.*
**Response:** Thank you for this comment. We agree that nest architecture, nesting behaviour and nest placement differ between these species. What we find fascinating is that we find clustering in bacterial assemblages between wet- and dry-nesters DESPITE these additional sources of variation. Although we would ideally be able to separate our analyses of dry-nesters further, as suggested by the reviewer, we hesitate to do so as this would further reduce our sample sizes, which are already relatively low. Separating our analyses further, or adding covariables to our existing analyses (e.g. nest architecture), will likely lead to the over-fitting of our models, leading to spurious results (see lines 201-202, 374 and 406-413 for a discussion on this).

The only species for which we can conduct further analyses is with the great-crested grebe (wet nester) and common coot (dry-nester), as the reviewer suggests. This additional analysis (lines 282-285 and S3 Table) confirm that eggshells of wet-nesters harbour a higher bacterial load than dry nesters, even when controlling for clutch size and sampling date.

Overall, we believe that the reviewer’s suggestions are great and indeed warrant further study, but is outside the scope of our current research. We now acknowledge the short-comings of our study, while continuing to highlight our main finding that nesting strategy appears to influence eggshell bacterial communities.

Discussion
**Comment 11:** *Line 364-366 – Sorry but this conclusion is too strong considering your sampling effort one to three nest per most of the dry-nesting species. Based on your dataset and fatal flaw with ignoring incubation phase in sampling egg, you cannot rule out the role of species identity on eggshell bacterial assemblage. Tone down this your conclusions and rather discuss thoroughly possible limitations of your study that more robust sampling controlled for nesting phase of sampling eggs in necessary to test these assumptions – i.e. rule out or test the effect of species identity on eggshell bacterial load. and discuss all these shortcomings of your study.*

**Response:** We have now toned down this statement, by removing the discussion on species identity and instead stating that “Overall, these results suggest that nesting strategy is an important determinant of bacterial assemblages and that conditions on egg surfaces may select for different bacterial assemblages”.

**Comment 12:** *Lines 388-391 (“Together, the moist nest conditions and likelihood of horizontal transmission could explain the higher loads across most bacterial taxa of wet-nesters compared to dry-nesters”) – also this conclusion needs to additional testing as I suggested. Use only dry-nesting species where appropriate number of individuals you have or cluster together ducks that have very specific nesting – incorporating of own body down feathers into the nest lining, egg covering and partial incubation. I think it will help to dilute all confounding effects that were omitted in your comparison and clustering all dry-nesting species in one group.*

**Response:** We apologise, but we are not sure what the reviewer is asking here. We are happy to respond to the reviewer if they further clarify their concern here.

**Comment 13:** *Former* *lines 336 –337 (“Interestingly, we generally found only minimal clustering of bacterial assemblages for species, especially for dry-nesting species. This is surprising, as the dry-nesting species differ markedly in their behaviour and ecology”) - of course if you sample one to three nest per species and sample only one egg per clutch. It is not representative eggshell microbial assemblage per species, sorry, but you need more robust sampling to see some nesting ecology-based clustering.*

**Response:** We have now removed this statement, as we agree that we cannot make strong conclusions about the influence specific species ecologies on eggshell bacterial assemblages. As previously mentioned, we now only focus only on our main result that wet-nesters have different bacterial assemblages to dry-nesters, and do not further explore species-specific effects.

**Comment 14:** *Former line 338 (“For example, ducks such as the mallard and red-crested pochard swim and forage in the water and therefore have intensive water contact”) – yes, and also cover their eggs with nesting material full of down feathers that may have thermoregulatory and also antimicrobial properties. Also intermitently incubate their eggs before starting full incubating which affect both, eggshell, but also egg white bacterial load. You should take into account also this very specific nesting ecology before choosing species for your study…or at least take this into account during testing your hypotheses and discuss these specifications thoroughly.*

**Response:** As discussed above, we have now removed statements such as these, as we agree we cannot make strong conclusions about the influence specific species ecologies on eggshell bacterial assemblages. We now only focus on our result that wet-nesters have different bacterial assemblages to dry nesters, and do not further explore species-specific effects.

**Comment 15:** *Former lines 343-344 and further (Despite these strong behavioural differences and the diverse taxonomic orders of these species, we detected no statistical clustering based on the degree of water contact in dry-nesting species. This suggests that nest microhabitat has a stronger influence on egg bacterial composition than bird phylogeny or ecology. ) – tone down thoroughly.. You really don´t have data on these strong conclusions.*

**Response:** As discussed above, we have now removed statements such as these, as we agree we cannot make strong conclusions about the influence specific species ecologies on eggshell bacterial assemblages. We now only focus on our result that wet-nesters have different bacterial assemblages to dry nesters, and do not further explore species-specific effects.

**Comment 16:** *Lines 411-413 – and also more thorough sampling of dry-nesting wetland species…*

**Response:** We agree. We have now added this statement to the text.

**Comment 17:** *Lines 432-437 – but there is also studies that did not find and effect and in general there is no study testing direct effect of trans-shell penetration and embryo mortality. Discuss more precisely. It is not such straightforward as you described. Most of eggshell bacterial are not able to live inside the eggs etc. etc…*

**Response:** Our text states that “Previous studies have shown…that experimental inoculation of eggs with eggshell-associated species increases embryonic mortality”. This is based on Hansen et al (2015) and we believe our statement to be true. In this study, the authors identified eggshell-associated bacteria and “inoculated isolates of Neisseria, Macrococcus, Streptococcus, and Rothia at various concentrations into developing chicken eggs. Seven-day mortality rates varied from 70 to 100%, depending on the bacterial species and inoculation dose”. Therefore, the study’s experimental inoculation of developing eggs from eggshell associated bacteria did indeed reduce mortality. We have also added an additional study (Cook et al 2005) which has shown that eggshell bacterial communities are associated with embryonic viability. Our literature search did not find a study finding that eggshell bacteria was **not** associated with embryonic viability. If the reviewer had a specific study in mind, we would be happy to include that in our text. We have, however, now also included a discussion highlighting that eggshell penetration and within-egg survival is influences by diverse egg-related factors (lines 435-436).

**Comment 18:** *Line 422 – yes, incubation, and how did you control this in your study??? Try to put this info into the context of your study. It is not discussion, just plain informative manner which you used here*.

**Response:** To conserve the flow of ideas in the paragraph the reviewer has highlighted, we prefer not to add additional speculation on the results that we may have obtained if we had included data on incubation in our analyses. We trust the reader recognises that the focus of this study is wet- vs dry-nesting, and that we cannot here explore the effects of egg incubation. We also highlight in the methods that we did not include additional co-variables in our analyses to reduce the probability of overfitting our statistical models (lines 201-202). In addition, the onset of incubation can be difficult to assess based on clutch size due to partial incubation (lines 201-212).

Overall, incubation is only mentioned in the Discussion as one possible mechanism which may explain the difference in bacterial assemblages between our water samples and eggshells of wet-nesters.

**Comment 19:** *Line 430-432 – your findings may highlight something but are not such robust, unfortunately. So try to tone down that your findings may serve as some initial stimuli for further more thorough testing*

**Response:** We have now toned down the original statement of “Our findings of differences in bacterial load and diversity between wet-nesting and dry-nesting species are significant…” to “Our findings **suggesting** differences in bacterial load and diversity between wet-nesting and dry-nesting species are significant…”.

**Reviewer 4**
**Comment 1:** *English proofreading is extremely recommended.*

**Response:** We have thoroughly reread the manuscript and made any additional grammatical improvements we deemed necessary. However, we are confident that our English in the manuscript is of a high standard, as 1) it was carefully read by one co-author who is a native English speaker and 2) no other reviewers commented on the English. Indeed, Reviewer 1 highlighted that “The progression from objectives and experimental methods to results and conclusions is exceptionally orderly and logically structured. This clarity enhances the significance of the research findings, emphasizing that the results are not merely speculative but hold meaningful evidence.”

**Comment 2:** *You should double check the references style according the submission guidelines.*

**Response:** We have followed the recommended PloSOne reference style throughout the article.

**Comment 3:** *Where are the references you followed for these experiments?*

**Response:** We used well-established techniques to investigate our study question, including bacterial culture and ARISA. We loosely based specific methods (e.g. how we swabbed egg surfaces) on our previous PLoSOne article on a similar topic (Brandl et al 2014), but modified the methods to suit the study question. To minimise extraneous content in the manuscript and given that we followed well-established protocols, we prefer not to elaborate further on this in the manuscript.

**Comment 4:** *What is the point of cultivating the samples on agar media that isn't selective?*

**Response:** As discussed in lines 162-163, using non-selective plates allowed the distinction between haemolytic and non-haemolytic bacteria. As haemolytic bacteria are known pathogens, this was an important distinction to make in our exploration on whether nesting-strategy increases the incidence of pathogenic bacteria on eggshells.

**Comment 5:** *For clarity, please provide a concluding paragraph at the end of the study.*

**Response:** Our original submission already included a concluding paragraph (lines 444-453) where we both summarise our main findings and offer additional avenues for future research.

**Comment 6:** *In order to increase clarity and conciseness, I would like to combine the discussion and results sections into a single section. This is because the sections are extremely disorganized and lack sufficient data.*

**Response:** Thank you for your suggestion. We respectfully disagree with your assessment and would like to highlight a comment by another reviewer regarding our manuscript, stating that “The progression from objectives and experimental methods to results and conclusions is exceptionally orderly and logically structured. This clarity enhances the significance of the research findings, emphasizing that the results are not merely speculative but hold meaningful evidence”. Additionally, all other reviewers agreed that the data presented were interesting and worthy of publishing. We believe these points underscore the overall quality and significance of our work and therefore prefer not to combine the Results and Discussion sections.

**Comment 7:** *Since you brought up the eggshells' antibacterial qualities, your study never touched this point.*

**Response:** The reviewer is correct that our study does not focus on the antibacterial qualities of eggshells. However, the key finding of our manuscript is that bacterial assemblages differed between dry- and wet-nesting species, suggesting that mechanisms exist that influence the persistence of eggshell bacteria. We therefore mention antibacterial properties of the shell as one such mechanism (e.g. lines 424-426, 435-436 and 449-450). Including possible mechanisms and explanations of research findings is a key component of Discussion sections, even when those mechanisms were not a key focus of the study. Indeed, we already recommend further study into antibacterial qualities of eggshells stating “further study on the nanostructural architecture of wet-nesting birds may well be illuminating” (lines 449-450).

**Comment 8:** *In order to make the results so compelling and connected to the clinical findings, it would also be helpful to discuss the most common disease in these species or the chicks after hatching.*

**Response:** A limitation of our study is that we do not know the exact identity of bacterial species quantified in our study (only broad taxa for the culture-based study, and not at all for the ARISA-based study). In addition, the epidemiology of our study species is very poorly known as they are all wild, and not commercial, bird species. It is therefore not possible to discuss specific diseases that affect adults and chicks of these species, and any discussions on disease needs to remain broad. We therefore only make general statements, such as “haemolytic bacteria, such as those sampled in this study and especially *Streptococcus*, are known pathogens and have been associated with diseases and mortality in birds” (lines 438-440). We acknowledge that substantial further research needs to be conducted on our study question before we can begin to answer questions on the potential for disease-causing bacteria to penetrate eggshells and influence embryonic survival in our study species.

**Comment 9:** *Additionally, I would advise outlining how susceptible these bacterial species are to antibiotics in order to determine whether they are commensal or dangerous infections (MDR pathogens), against which certain bird species are immune. (Multidrug-resistant (MDR) pathogens are bacteria that are resistant to multiple antibiotics. They are a serious threat to public health and can cause severe infections).*

**Response:** Thank you for this comment. However, discussions of multidrug-resistant pathogens are beyond the scope of the study and not entirely relevant to these wild species that normally do not receive antibiotics. As discussed above, we do not know the identity of the species we have sampled, beyond broad taxa (e.g. *Enterococcus* species). Even strains within the same bacterial species are known to present different pathogenicity and resistance to antibiotics. We therefore prefer not to speculate and instead focus our discussions on our main findings.

**Comment 10:** *Why did you limit your attention to bacteria? How about fungi?*

**Response:** Further research on fungal infection of eggs is a great idea, but outside scope of current study. The two techniques used here (bacterial culture and ARISA) have both been developed for bacteria and cannot be simultaneously be used to sample fungi. We believe that our focus on only bacteria, using two complementary techniques, allows us to make novel findings on the role of nesting strategy on eggshell microbiomes in wetland-associated species.

**Comment 11:** *Where is the raw data for the genetic findings?*

**Response:** PLOSOne guidelines require the upload of raw data used to generate 1) the values behind the means, standard deviations and other measures reported and 2) the values used to build graphs. We have therefore now uploaded this data with our resubmission.

**Comment 12:** *Fig 1: the error bars are so weird, please explain it.*

**Response:** We apologise, but we do not understand the issue that the reviewer has with the error bars in Figure 1. As already stated in the figure captions, the graphed data represent mean logarithmic abundance **(±SE)** of colony-forming units grown from eggshell bacterial swabs from the thirteen study species. If the reviewer could further clarify the information they require, we would be happy to provide this.

**Comment 13:** *Please divide Table 1 into dry and wet categories with distinct data for each since it is worthless. Please explain what t/p stands for as well.*

**Response:** Thank you for your comment. The table is indeed already divided into wet and dry nests. “Coefficient (dry/wet)” provides information on the direction of any significant differences in our data, with more negative values corresponding to higher bacterial loads on wet nests (as the coefficients for wet nests were set to zero). In addition, t and p are two well-established statistical terms. As the three other reviewers had no issue with this table, we prefer to leave it in its original form.

**Comment 14:** *Line 23: Be specific and use birds instead of animals.*

**Response:** The content in the beginning of Abstracts and Introductions is typically broad and then becomes more specific as they progress. We therefore firstly discuss bacteria in the context of **all** animals (e.g. we mention bacteria on both skin and feathers in the Introduction) and then narrow our discussions to birds. We therefore prefer to use “animals’ instead of “birds”.

**Comment 15:** *Line 79: Bacterial first, then genetic.*

**Response:** Thank you. We have made this change.

**Comment 16:** *Line 196: Please provide an illustrative figure to demonstrate the steps taken in a sequential manner, as the sampling numbers are extremely unclear.*

**Response:** We are unclear how an illustrative figure can clarify sampling numbers. We already state detailed sample numbers in lines 102-118, highlighting the thirteen species we used for the study, and the number of samples taken from each species. These samples were used for both the bacterial culture and ARISA. The exact number of samples used in each analyses differs markedly, depending on the nature of the analysis. For example, we conduct 1) GLMM for all species, 2) GLMM for coots vs great-crested grebe, 3) PERM for water by location, 4) PERM for wet- vs dry-nesters, 5) GLMM for similarity between water and wet- and dry-nesting eggs, amongst many other analyses. Creating an illustrative figure for these many combinations of sample sizes would be highly complex and convoluted, and we therefore prefer not to include one here.

**Comment 17:** *Throughout the manuscript, use the same first line space.*

**Response:** We are not entirely clear to what the reviewer is referring to, but assume that they are referring to line indentation. We have used the consistent formatting approach of not indenting the first line of each section, but then indenting the remaining paragraphs. If the reviewer was referring to a different aspect of the first line space, please let us know and we will respond accordingly. In any case, we are confident that, if our formatting is incorrect, it will be corrected during the type-setting stage of publication production.

**Comment 18:** *L235: Findings? For clarification, please specify which figure.*

**Response:** We now state “see nMDS analyses in Results”.

**Comment 19:** *L276-279: For wet or dry nesting samples, is 35.2%?*

**Response:** We already state in line 276 that these data are “across all samples”.
