# Supplementary material for: Bacterial assemblages on eggs reflect nesting strategies in wetland-associated birds
Source: PLoS One. 2025 Sep 17;20(9):e0332380. doi: 10.1371/journal.pone.0332380 (PMC12443268; doi:10.1371/journal.pone.0332380)
Supplement: Attachment — Submitted filename: Response_to_reviewers_auresp_2.docx [file pone.0332380.s012.docx]

**Academic Editor
Comment 1:** *While the introduction and discussion mention vertical transmission, the study does not provide data to distinguish it from environmental acquisition. This limits conclusions about maternal contribution to eggshell microbiota.*

We agree that we cannot make firm conclusions on the maternal contribution to egg shell microbiota, nor tease apart vertical versus horizontal effects. Considering this caveat, we have modified the first paragraph in the discussion to better highlight this (lines 364-369), where we now state that “Our data do not allow us to robustly distinguish the contributions of horizonal and vertical transmission on the assemblage characteristics of eggshell-associated bacteria in our study. However, the association between nesting strategy and eggshell bacterial communities suggest that environmental sources may be a significant contributor of eggshell bacteria in our study species”. We hope that this statement sufficiently emphasises this limitation of our study.

**Comment 2:** *Nesting strategy (wet vs. dry) is used as a broad grouping variable, but species within each group vary significantly in nesting materials, water contact, and incubation behavior. This undermines the strength of clustering conclusions based on nest type alone.*

Thank you for this comment, which we agree with. We discuss this limitation in detail in detail in the Discussion (lines 381-388), concluding that “Future studies would benefit from including more wet-nesting species and more individuals per species. This will allow a more detailed and robust exploration of the determinants of fine-scale variation in bacterial abundance, such as types of nesting materials used, incubation behaviour and temporal influences on microbiota. Additionally, sampling more than one egg per clutch would be beneficial to account for intra-clutch variation in eggshell microbiota (e.g. due to egg laying order, partial incubation or brood parasitism), while using candling techniques to estimate embryo age within each egg would help account for microbial variation due to incubation stage.”

In addition, our concluding paragraph (lines 466-468) now states that “Future studies would benefit from increased sample sizes, both within and across species, to allow a robust exploration of not only nesting strategy on eggshell microbiota, but other ecological factors such as egg laying date, incubation, and nesting materials”.

**Comment 3:**  *One Egg per Nest Sampled**: Sampling only one egg per clutch does not account for intra-clutch variation, which can be substantial and could bias results - discuss this in the Limitations section.*

Thank you for this comment. In lines 384-388 we now state that “Additionally, sampling more than one egg per clutch would be beneficial to account for intra-clutch variation in eggshell microbiota (e.g. due to egg laying order, partial incubation or brood parasitism), while using candling techniques to estimate embryo age within each egg would help account for microbial variation due to incubation stage.”

**Comment 4:** *Bacterial communities change with incubation, and the study did not assess incubation stage or egg laying order. Without controlling for this, variability may mask true differences due to nesting strategy - discuss this in the Limitations section.*

As mentioned above, we now acknowledge that a limitation of our study is our small sample sizes, which meant that we could not control for additional ecological factors such as incubation behaviour, brood parasitism, and temporal patterns, which indeed could mask true differences due to nesting strategy.

**Comment 5:** *Many dry-nesting species have sample sizes of 1–3 nests, making any comparison across species or nesting strategies tenuous - discuss this in the Limitations section.*

Yes, we agree this is a limitation of our study. We have slightly modified the limitations paragraph to now state that “Our sample sizes are higher for dry-nesters (eleven species), while we only include two wet-nesting species in our analysis, due to the limited number of wet-nesting species in the study area. In addition, samples sizes within species were often limited. This restricts the range of analyses that we can perform on our data, and could mask true differences in bacterial assemblages with nesting strategy” (lines 375-379).

**Comment 6:** *Use of Outdated Genetic Method (ARISA). While ARISA is a valid fingerprinting technique, it has been largely supplanted by 16S rRNA sequencing and shotgun metagenomics, which offer finer resolution. The authors acknowledge this but should clearly frame ARISA's limitations.*

We have now updated the description of ARISA to better frame its limitations, especially in the context of metagenomics and other more-modern techniques, stating that “Nonetheless, ARISA can still only provide an estimate of diversity due to issues associated with taxon-specific differences in genomic structure (e.g. some species do not have the 16S and 23S genes organised in an operon), leading to some bacteria being undetected and others over-represented….Despite recent advances in the molecular characterisation of bacterial communities, which allow finer resolution of bacterial assemblages (e.g. metagenomic techniques), ARISA and culture-based techniques remain effective tools to characterise broad differences in assemblages between treatment groups” (lines 136-145).

**Comment 7:** *Avoid Overstating Conclusions. Many conclusions regarding the effect of nesting strategy are too strong given the limitations in design. These should be reworded to reflect the exploratory nature of the study.*

We have reread our manuscript and softened our concluding statements, to better reflect the exploratory nature of the study.

**Comment 8:** *Clarify Figures. Figures should include clearer legends and visual indicators (e.g., nesting strategy symbols or species groupings). Consider separating duck species from other dry-nesters in visualizations.*

Thank you for this comment. We feel that the figure which could best use visual indicators for nesting strategies is Figure 2 (nMDS results for bacterial assemblage vs species/nesting strategy). This figure however already includes visual indicators (circles for dry nesters and triangles for wet-nesters). Although we like the idea of separating ducks from other dry-nesters in visualisations, we feel that this will overcomplicate the figure with too many symbol-types. Instead, the reader can identify each species by the colour of each symbol and by reading the key in the figure caption. We therefore prefer to leave the figure unmodified.

**Comment 9:** *Specify that future research should explore egg antimicrobial traits, control for incubation stage, and incorporate 16S sequencing to identify microbial taxa involved in pathogenic vs. commensal roles.*

We now conclude our Discussion by stating that “Future studies would benefit from increased sample sizes, both within and across species, to allow a robust exploration of not only nesting strategy on eggshell microbiota, but other ecological factors such as egg laying date, incubation, and nesting materials. Moreover, future research should adopt more advanced molecular approaches, such as 16S rRNA gene sequencing and metagenomics, to enable deeper and more accurate characterisation of microbial communities” (lines 466-471).

**Comment 10:** *Line 23: Change “Animals host diverse bacterial assemblages” to “Birds host diverse bacterial assemblages” to reflect study focus.*

We have made the suggested change.

**Comment 11:** *Line 168: Add “the” before “media”: “…onto the media.”*

We have made the suggested change.

**Comment 12:** *Hyphenation: Standardize the use of “wet-nesting” and “dry-nesting” throughout the manuscript.*

We have revised the consistent use of “wet-nesting” and “dry-nesting” throughout the manuscript. Broadly, we use the term "wet-nesting" as an adjectival descriptor of nesting strategy, while the noun phrase "wet nest" describes the physical structure itself. This distinction helps clarify whether we are referring to the actual nest or to the broader nesting strategy.

**Comment 13:** *Tone Down Phrasing: Replace strong language such as “our findings are significant” with “our findings suggest” or “indicate.”*

We have revised to manuscript to soften our conclusions throughout. In terms of your specific comment here, the statement we believe you are referring to is: “Our findings suggesting differences in bacterial load and diversity between wet-nesting and dry-nesting species are significant, as trans-shell penetration of bacteria can occur, which can increase embryonic mortality.” We have not modified this sentence as we state that our findings SUGGEST a difference and this possible difference WOULD be significant for the reasons described.

**Comment 14:** *Line 196: Improve clarity of sampling description—consider a flowchart or table to summarize sample numbers.*

In lines 116-121, we now state that “In total, we sampled 101 egg and 24 water samples (total 125 samples). All samples successfully produced cell cultures (i.e. total sample size of 125 samples). In contrast, the success of our DNA extraction for the genetic characterisation of microbiota was more variable, resulting in reduced sample sizes for these analyses. This corresponded to 73 egg and 22 water samples for all genetic analyses. See S2 Table for further details”.

Please note that, during our revision of sample sizes, we identified a minor error in the reported number for the across-egg similarity analyses (lines 336 and 341), which was initially stated as *N* = 82 (instead of N = 73). This discrepancy was limited to the reported value and did not affect the underlying analysis. To ensure consistency and accuracy, we reviewed sample sizes across all other analyses and confirmed that no further issues were present.

**Comment 15:** *Phrase such as “highlight important differences” should be reworded to “suggest potential differences.”*

Thank you. As stated above, we have now reread the manuscript and toned down these strong statements.

**Comment 16:** *Ensure consistent italicization of all Latin species names throughout the manuscript, including the References.*

We have revised the manuscript and made any relevant changes. Please note that ‘coliforms’ is not a genus (or species) name and is therefore no italicised.

**Comment 17:** *The manuscript references older papers for methods but does not cite newer alternatives (e.g., 16S sequencing), which should at least be discussed.*

We now mention metagenomic techniques in the Methods (line 143), and also provide several references to more modern genetic techniques in the Discussion (lines 469-470).

**Reviewer 3**

**Comment 1:** *Why you changed corresponding author in the revised version of the manuscript and why one of the co-authors (L. Rubáčová) has been removed from the author team to Acknowledgements only? Did she agree with this and it was discussed with her thoroughly? It is not usual such dramatically change author membership and status of authors?*

The corresponding author was changed in consultation with PloSOne and was a necessity to allow for payment for the publication of this manuscript. Despite this change, all co-authors were involved in the revisions of the manuscripts.

 L. Rubáčová contributed to the acquisition of the data used in this manuscript. While we initially included her as a co-author in recognition of this contribution, she later expressed a preference to be acknowledged rather than listed as an author, feeling that her involvement did not meet her personal threshold for authorship. We respect and appreciate her perspective and have honoured her request by acknowledging her contribution accordingly.

**Comment 2:** *To your comments regarding clutch age (my (Reviewer 3) Comment 1) and effect on eggshell bacterial assemblage. Yes, you considered sampling date and describe that effect might be linked with changed environmental factors. But, I am considering incubation of the clutch and resulting eggshell assemblage. As you described on lines 150-152, you sampled one egg from nest when you found it, but although you record the precise date of nest finding, you were not aware of the age of the clutch (i.e. how long the clutch has been incubated already). Moreover, it is quite common that 1) nesting parasitism occurred in Mallards and also coot and grebes and also that 2) intermittent incubation of partial not-completed clutch occurred before the full incubation and clutch completion (at least in ducks it is very common, and I think also swans or grebes are known for this behaviour). In practice, simple fieldwork “candler” (inspection of egg inner structure under the light) is used for determination of embryo age which correspond to intensity of incubation. And as you mentioned in you text of the revised ms that incubation may affect eggshell bacterial assemblage (and quite a lot), this discrepancy in eggs age and developmental stage within the clutch may cause bias if your chosen only one egg from the complete clutch for sampling. I think you should somehow touch of this possible bias in your data caused by the different clutch age, role of intermittent incubation and/or brood parasitism in your focal species.*

Thank you for this further clarification. We have now discussed this in more detail in lines 384-388, considering the reviewer’s comments, stating that “Additionally, sampling more than one egg per clutch would be beneficial to account for intra-clutch variation in eggshell microbiota (e.g. due to egg laying order, partial incubation or brood parasitism), while using candling techniques to estimate embryo age within each egg would help account for microbial variation due to incubation stage.”

**Comment 3:** *But in general, it is nice you added additional GLMMs testing for the effect of clutch size and sampling date, as it is really highly probably that enlarged clutches may be incubated before clutch completion or being parasitized by brood parasites. This additional GLMMs made your conclusions more robust. Yet, still, as your study is pioneering in study of bacteria on eggshell with respect to breeding ecology, I am for to include possible factors that might be behind the differences between dry- and wet-nesters. Although I understand your point of view and you want to keep the line of the study as simple as possible, I think a few sentences in the discussion with other potential factors cannot be distracting from the major message of your study.*

Thank you. As mentioned above, we have now added discussion of other possible factors behind the differences in wet- and dry-nesters (e.g. lines 381-388 and 407-416).

**Comment 4:** *Line 141 -* *“more holistic overview”., I suggest to replace holistic by “comprehensive”. Holistic is really to strong meaning in your case study.*

Suggested change made.

**Comment 5:** *Line 142 - …in isolation. I suggest to replace by “separately” or some other equivalent.*

Suggested change made.

**Comment 6:** *Line 286-287 – “….of bacteria on eggshells on wet-nesting..“ rephrase it please.*

Suggested change made

**Comment 7:** *Line 296 – “coliforms” without capital please*

Suggested change made

**Comment 8:** *Lines 359-366 – I am still wondering if it cannot be partially caused by pre-incubation behaviour of dry-nesters – covering of clutch by down feathers in mallard and other duck species, intermittent incubation I have mentioned above. I think you should take this into account in discussion of your results (mostly differences between dry and wet-nesters). You already did for covering the clutch by feathers, and even if you disagree to do for incubation (see response to my Comments 18). I think it can help to untangle the all possible confounding factors that have to be taken into account in future studies of eggshell or trans-shell bacterial assemblages.*

Thank you for this comment. As explained above, we have now acknowledged the potential role of factors such as partial incubation and brood parasitism on differences between wet- and dry-nesters (lines 384-388). We also stated that “Finally, some species may display intermittent incubation or experience brood parasitism, which can further complicate interspecies explorations of eggshell-associated bacteria” (lines 414-416).

**Comment 9:** *Line 372 The aim of this study…..I suggest to rephrase… “…bacterial abundance and community structure vary based on……”.*

Suggested change made

**Comment 10:** *Your Response to Comment 17 of Reviewer 3 – for example this study has show no effect of bacterial trans-shell penetration on hatching success: Effect of intermittent incubation and clutch covering on the probability of bacterial trans‐shell infection - Javŭrková - 2014 - Ibis - Wiley Online Library.*

Thank you for highlighting this study, which we have now included in our discussion on eggshell penetration by bacteria (lines 448-449).
