# Supplementary material for: Bacterial assemblages on eggs reflect nesting strategies in wetland-associated birds
Source: PLoS One. 2025 Sep 17;20(9):e0332380. doi: 10.1371/journal.pone.0332380 (PMC12443268; doi:10.1371/journal.pone.0332380)
Supplement: Attachment — Submitted filename: Response_to_reviewers_auresp_3.docx [file pone.0332380.s013.docx]

**Academic Editor
Comment 1:** *The conclusions still extend beyond what the data can support. Several sections of the abstract and discussion state or imply that wet-nesting results in higher bacterial loads and that this has consequences for embryonic mortality. This study is observational and does not test cause and effect or hatching outcomes. These implications should be presented as hypotheses for future research rather than results supported by the current data. In particular, the discussion around lines 443 to 444 and the associated summary sentences in the abstract need to be reworded to reduce the strength of the claims.*

**Response:** We have rewritten sections of the Abstract and Discussion to present our interpretations more cautiously. These changes highlight that our data suggest an intriguing link between nesting strategy and eggshell-associated bacteria, but that more comprehensive sampling, with more advanced genetic techniques, are required to confirm these patterns (e.g. lines 39-42, lines 368-371 and 463-474).

In addition, we have greatly reduced our discussion on trans-shell penetration in the Discussion section. We agree that it is too early to make detailed speculation on the consequence of nesting strategy in the study species on embryonic survival. We now only briefly mention this as a cautious possibility in the penultimate paragraph of the Discussion (lines 455-462)

**Comment 2:** *The categorization of wet-nesting versus dry-nesting species is too broad. This point was acknowledged but the language in the results and discussion still reads as if general conclusions across these categories can be made. The sample of wet-nesting species is restricted to two closely related species and the dry-nesting group is heterogeneous in water contact and nest materials. Each time the results are generalized across these categories, an explicit caveat about these limitations should be included.*

*(In addition), the possibility that phylogeny influences bacterial clustering has not been addressed with sufficient emphasis. Both wet-nesting species belong to the same family. Clustering patterns could therefore reflect shared evolutionary history rather than nest characteristics. The discussion should explicitly highlight that these factors cannot be separated in the present dataset and that the effect of nesting strategy should be interpreted with caution.*

**Response:** We have now revised our text to better clarify the limitations of our data, in particular the close-relatedness of the two wet-nesting species and the broad phylogeny of the dry-nesting species. We now consistently state that the wet-nesting species are closely-related, while our category of dry-nesting is broad (e.g. lines 106-111, lines 361-364 and 410-412). The paragraph in lines 410-432 continues to discuss the fact that the study requires an increase in species in both categories to make definitive conclusions on nesting strategy and eggshell bacteria. We also discuss the fact that the dry-nesting category is very broad and includes species with very different ecologies and behaviours, which further complicates the interpretation of our data. We have added a sentence stating that “our nMDS analyses suggested no obvious clustering of bacterial assemblages of the family Anatidae, which includes ducks, geese and swans, despite their unique nesting behaviours (e.g. intermittent incubation)” (lines 421-423). This latter point is illustrated in Figure 2, where we have updated the graph symbols to clearly indicate which species belong to the Anatidae family.

**Comment 3:** *The clarity of the figures remains an issue. Figure 2 in particular is difficult to interpret due to overlapping symbols. Although the legend provides color codes, the visual separation of species is poor. Symbols could be made partially transparent, larger, or shown in separate panels for wet and dry nesters. The figure caption should also report the stress values for the multidimensional scaling. These were previously requested but have not been implemented.*

*(In addition), one of the earlier reviewer requests to visually separate ducks from other dry-nesting species in the figures was declined by the authors. If this decision is retained, the text should guide the reader through the interpretation of symbol codes more explicitly so that the influence of ducks on clustering patterns can be evaluated by readers.*

**Response:** We have revised Figure 2 in response to concerns about its interpretability. The updated figure features larger, semi-transparent, and more distinct, symbols to enhance visual differentiation among species. Additionally, a visual legend has been added at the bottom of the figure to clearly associate each symbol with its corresponding species.

Members of the Anatidae family (swans, ducks, and geese) are now highlighted using open circles. This modification more clearly demonstrates a likely lack of clustering of bacterial assemblages of the family Anatidae, despite their unique nesting behaviours. This is now also mentioned in the Discussion (lines 421-423).

Finally, we have added the stress value for the nMDS in the figure caption, which was unintentionally missed in previous revisions of this manuscript. We hope these modifications improve the clarity and usefulness of the figure.

**Comment 4:** *The description of patterns in bacterial similarity between eggs and water could be misinterpreted. The text attributes differences mainly to ecological filtering, yet differences in the sampling methods and the inherent biases of ARISA are also plausible explanations. These limitations should be clearly distinguished from ecological interpretations.*

**Response:** Yes, we agree that the water data should be interpreted with caution. As noted in lines 274–282 of the Methods, the differing approaches used to sample eggshells and water constrain the conclusions that can be drawn when comparing egg-associated and water-associated bacteria. Accordingly, we have already limited the scope of our analyses involving water-associated bacteria.

In addition, we have now modified the text in the Discussion on the limitations of the analyses with the water-associated bacteria. We stress that, as the Bray-Curtis dissimilarity index used in the nMDS incorporates both OTU identity and abundance, field and genetic sampling issues may obscure true variation in bacterial assemblages associated with nesting strategy (lines 433-444). We then continue by discussing the possible ecological explanations for the observed dissimilarity between eggshell-associated and water-associated bacteria.

**Comment 5:** *The study relies on ARISA and culture-based methods, and while this is acceptable for exploratory work, these approaches provide low resolution. The methods section and the discussion mention these limitations, but they should be restated more clearly in the abstract and conclusion. This would help to place the findings in the context of recent research that uses higher resolution molecular approaches.*

**Response:** We have now added additional text to both the abstract and conclusion to highlight that, while our results are promising, further research with more advanced molecular approaches are required. Specifically, in the Abstract we state “Further research is, however, required to confirm these patterns, incorporating more comprehensive sampling and utilising more advanced genetic approaches” (Lines 41-42). In addition, the Conclusion now states that “future research should adopt more advanced molecular approaches, such as 16S rRNA gene sequencing and metagenomics, to enable a deeper and more accurate characterisation of microbial communities” (lines 472-474).
